# Kuafuorterviruses, a novel major lineage of reverse-transcribing viruses

**DOI:** 10.1093/ve/veae110

**Published:** 2024-12-19

**Authors:** Zhen Gong, Guan-Zhu Han

**Affiliations:** College of Life Sciences, Nanjing Normal University, Nanjing, Jiangsu 210023, China; College of Life Sciences, Nanjing Normal University, Nanjing, Jiangsu 210023, China

**Keywords:** reverse transcribing virus, retrotransposons, virus evolution, transposable elements

## Abstract

Reverse-transcribing viruses (RTVs) characterized by reverse transcription required for their replication infect nearly all the eukaryotes. After decades of extensive analyses and discoveries, the understanding of the diversity of RTVs has largely stagnated. Herein, we discover a previously neglected lineage of RTVs, designated Kuafuorterviruses, in animals. Through screening over 8000 eukaryote genomes, we identify the presence of endogenous Kuafuorterviruses in the genomes of 169 eumetazoans dispersed across 11 animal phyla. Phylogenetic analyses and sequence similarity networks indicate that Kuafuorterviruses constitute a novel major lineage of RTVs. The discovery of Kuafuorterviruses refines our understanding of the diversity, evolution, and classification of RTVs and has implications in annotating animal genomes.

## Introduction

Reverse-transcribing viruses (RTVs; the kingdom *Paranavirae*), which require reverse transcription during their replication, can infect nearly all the eukaryotes (Ahlquist 2006). RTVs include many pathogens of relevance to public health and agriculture, such as “human immunodeficiency virus,” “hepatitis B virus,” and “cauliflower mosaic virus.” To date, six families of RTVs have been officially defined by the International Committee on Taxonomy of Viruses, namely, *Metaviridae, Pseudoviridae, Belpaoviridae, Retroviridae, Caulimoviridae*, and *Hepadnaviridae* (Krupovic et al. 2018). While *Hepadnaviridae* stands as the singular family within the *Blubervirales* order, the remaining five families are unified into the *Ortervirales* order (Walker et al. 2020). *Metaviridae, Pseudoviridae, Belpaoviridae*, and *Retroviridae* possess dual features of transposons and viruses and are also known as the four superfamilies of long terminal repeat (LTR) retrotransposons, namely, Ty3/Gypsy, Ty1/Copia, and Bel/Pao retrotransposons and endogenous retroviruses, respectively (Wicker et al. 2007). Endogenous RTVs comprise a major component of the eukaryote genomes, substantially contributing to the evolution of eukaryote genome complexity (Wicker et al. 2007). Recently, two new families of RTVs closely related to retroviruses, tentatively termed as *Lokiorterviridae* and *Odinorterviridae*, have been discovered through phylogenomic analyses (Wang and Han 2021, 2022), implying the presence of novel major lineages of RTVs.

In this study, we report the discovery of a previously neglected lineage of RTVs, designated Kuafuorterviruses, in numerous animal genomes. Phylogenetic analyses and sequence similarity networks (SSNs) indicate that Kuafuorterviruses constitute a novel major lineage of RTVs. Our findings refine the understanding of the diversity, evolution, and classification of RTVs.

## Materials and methods

### Identification of Kuafuorterviruses in eukaryote genomes

We conducted similarity search combined with phylogenetic analyses to identify Kuafuorterviruses within 8001 eukaryote genomes (including 1029 protists, 790 plants, 2772 fungi, and 3410 animals). First, we employed the tBLASTn algorithm to search against eukaryote genomes with the reverse transcriptase (RT) protein (XP_035688522.1) as the query and an *e* cutoff value of 10^−5^. XP_035688522.1 was identified during mining and annotating retrotransposons within the genome of *Branchiostoma floridae* using HMMER. Next, phylogenetic analyses of significant hits, representative retroelements, and Kuafuorterviruses were performed using an approximate maximum likelihood method implemented in FastTree (Price et al. 2010). Alignments were generated using the L-INS-I method implemented in MAFFT (Katoh and Standley 2013). Sequences that clustered with Kuafuorterviruses were retrieved for further phylogenetic analyses. Complete Kuafuorterviruses with 5ʹ- and 3ʹ-LTRs were identified using LTRharvest with default parameters (Ellinghaus et al. 2008). Genomic architectures of Kuafuorterviruses were annotated using the hmmscan method implemented in HMMER with default settings (http://hmmer.org/). Structure prediction of putative Gag proteins was conducted using Phyre2 (Kelley and Sternberg 2009) and HHpred (Zimmermann et al. 2018).

### Evolutionary dynamics of Kuafuorterviruses

We retrieved all the Kuafuorterviruses with 5ʹ- and 3ʹ-LTRs from three representative animal species (*Gigantopelta aegis, Morbakka virulenta*, and *Pachyseris speciosa*) to explore the evolutionary dynamics of Kuafuorterviruses within these species. LTR sequences were aligned using the L-INS-I method implemented in MAFFT (Katoh and Standley 2013). The genetic distance between 5ʹ-LTR and 3ʹ-LTR was calculated using the Kimura two-parameter substitution model.

### Phylogenetic analyses of RT proteins

To explore the phylogenetic relationships between Kuafuorterviruses and representative retroelements that cover all the diversity of known retroelements, we performed phylogenetic analyses of the RT domain. The alignment was generated using the L-INS-I method in MAFFT (Katoh and Standley 2013) and refined manually. Phylogenetic analysis was performed using a maximum likelihood method implemented in IQ-TREE (Nguyen et al. 2015). The best-fit substitution model was selected using the ModelFinder algorithm (Kalyaanamoorthy et al. 2017). Supports for the nodes were assessed using the ultrafast bootstrap (UFBoot) approach with 1000 replicates (Hoang et al. 2018).

To further explore the phylogenetic relationships between Kuafuorterviruses and LTR-like retroelements, we performed phylogenetic tree analyses and phylogenetic network analyses based on two RT protein datasets, namely, small (80 elements) and large (418 elements). LTR-like retroelements in both datasets were selected based on large-scale phylogenetic analyses covering the major diversity of known retroelements. Alignments and phylogenetic trees were generated using the aforementioned approach. Phylogenetic networks for both datasets were performed based on uncorrected_P distances using the NeighborNet algorithm implemented in SplitsTree 4 (Huson and Bryant 2006).

### SSN reconstruction

We reconstructed SSNs based on the genetic distance of RT proteins for both small and large datasets. For visualization, we used the Fruchterman–Reingold layout in Gephi (Bastian et al. 2009) with 1/distance as the edge weight and filtered edges below 0.666. To resolve cluster composition, we used two algorithms, the modularity calculation (MC) and the connected component (CC) statistic, implemented in Gephi. Rand index, which quantifies the similarity between two different clustering schemes, was accessed using flexclust package in R.

## Results

### Kuafuorterviruses are widespread in animals

**Figure 1.**
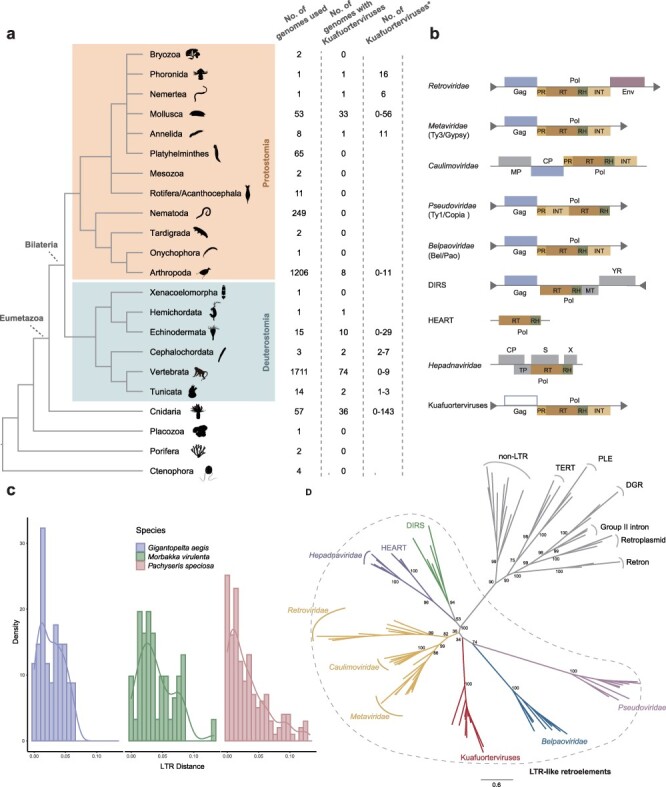
The distribution and feature of Kuafuorterviruses. (a) The distribution of Kuafuorterviruses within the animal genomes. The phylogenetic relationship of metazoans is based on the literature (Telford et al. 2015, Li et al. 2021). Animal silhouettes are retrieved from PhyloPic (http://www.phylopic.org/). The numbers of genomes used in this study and the numbers of genomes in which Kuafuorterviruses were identified based on RT proteins are shown next to the species. The asterisk indicates the copy number of Kuafuorterviruses with two flanking LTRs in the species that possess Kuafuorterviruses based on mining RT proteins. (b) Classical genomic architectures for LTR-like retroelements. LTRs and terminal inverted repeats are represented by gray triangles in the same orientation and different orientation, respectively. Abbreviations: CP, capsid protein; Env, envelope protein; MP, movement protein; MT, DNA N6-adenine-methyltransferase; S, surface protein; TP, terminal protein; YR, tyrosine recombinase. (c) Evolutionary dynamics of Kuafuorterviruses within three species. The histograms and density curves indicate the distribution of the genetic distance between 5ʹ- and 3ʹ-LTRs. After the integration of an RTV, the genetic distance between its 5ʹ- and 3ʹ-LTRs increases over time. (d) Phylogenetic relationships of RT proteins from representative retroelements. The phylogenetic tree was inferred based on the RT protein sequences using a maximum likelihood method. UFBoot support values are shown near selected nodes.

Initially, we identified a novel lineage of RTVs that cannot be readily classified based on the up-to-date RTV classification system during mining LTR retrotransposons within the genome of *B. floridae*. We designated this lineage of RTVs “Kuafuorterviruses,” which is named after a legendary figure from Chinese mythology. Then, we leveraged a large-scale phylogenomic approach to screen Kuafuorterviruses within the genomes of 8001 eukaryotes, including 1029 protists, 790 plants, 2772 fungi, and 3410 animals. We identified the presence of endogenous Kuafuortervirus in the genomes of 169 eumetazoans that are dispersed across the major diversity of animals (11 out of 23 animal phyla) based on mining RT proteins (Fig. 1a; Supplementary Table S1). Copy numbers of complete endogenous Kuafuorterviruses with two flanking LTRs vary greatly across animal groups, ranging from 0 to 143. Overall, endogenous Kuafuorterviruses appear to be relatively rare in vertebrates but abundant in cnidarians (Fig. 1a; Supplementary Table S2). Note that for most of the animal phyla, only several to tens of species have been sampled and sequenced for their genomes and Kuafuorterviruses might be more widely distributed in animals. Yet, no Kuafuortervirus was identified in eukaryotes outside animals except one protist, *Nephromyces* sp. ex *Molgula occidentalis*, in which Kuafuorterviruses might have derived from horizontal transfer or sequencing contamination because the protist is an endosymbiont associated with molgulid ascidian tunicates (Munoz-Gomez et al. 2019). Nevertheless, our results indicate that Kuafuorterviruses are widespread in the genomes of animals.

### Kuafuorterviruses are actively proliferating in animal genomes

In general, the genome architectures of Kuafuorterviruses are similar to those of known RTVs (Fig. 1b; Supplementary Dataset S1). Kuafuorterviruses are flanked with LTRs at 5ʹ- and 3ʹ-termini and encode polymerase polyprotein (Pol) with protease, RT, RNase H, and integrase domains (Fig. 1b; Supplementary Table S3). The length of a majority of Kuafuorterviruses is between 5000 and 6000 nucleotides. There is a long region between 5ʹ-LTR and Pol potentially coding Gag protein, which shares structural similarity with known Gag proteins (Fig. 1b). Some domains, such as Exo_endo_phos and TetR_C_24, are only present sporadically in Kuafuorterviruses, which may be derived from hosts (Supplementary Table S3). Moreover, the YXDD motif in canonical RT enzymes is present as IADD, VADD, or LADD in the RT domains of Kuafuorterviruses (Sharma et al. 2005). To explore the evolutionary dynamics of Kuafuorterviruses, we analyzed three representative species, *G. aegis* (Mollusca), *M. virulenta* (Cnidaria), and *P. speciosa* (Cnidaria). A total of 143, 49, and 44 complete Kuafuorterviruses with 5ʹ- and 3ʹ-LTRs were identified in *P. speciosa, G. aegis*, and *M. virulenta*, respectively. The distribution of the genetic distance between 5ʹ- and 3ʹ-LTRs indicates that Kuafuorterviruses are still actively proliferating in these three species (Fig. 1c).

### Kuafuorterviruses constitute a novel major RTV lineage

**Figure 2.**
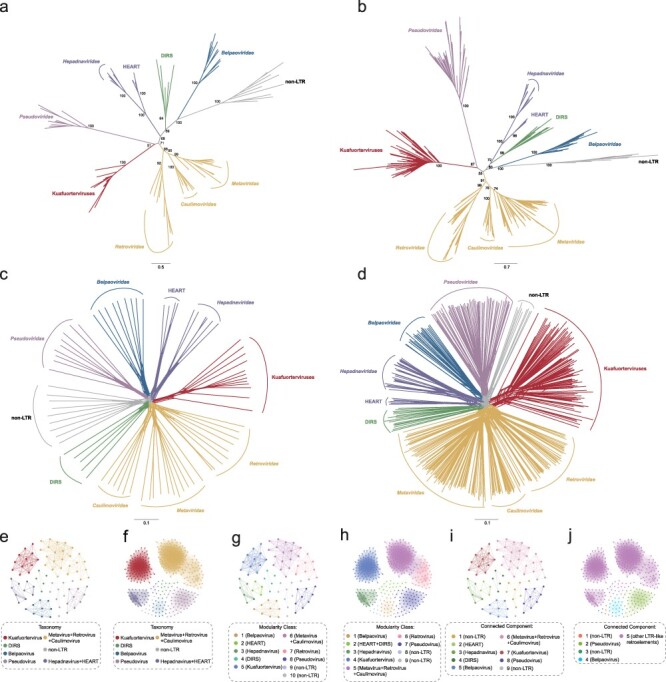
Evolutionary relationships of Kuafuorterviruses and known LTR-like retroelements. (a), (c), (e), (g), and (i) are for the small dataset, whereas (b), (d), (f), (h), and (j) are for the large dataset. (a and b) Phylogenetic trees of Kuafuorterviruses, known LTR-like retroelements, and non-LTR retrotransposons. The phylogenetic trees were inferred based on RT proteins using a maximum likelihood method with non-LTR retrotransposons as outgroups. UFBoot support values are shown near the selected nodes. (c and d) Phylogenetic networks of Kuafuorterviruses, known LTR-like retroelements, and non-LTR retrotransposons. The phylogenetic networks were reconstructed based on RT proteins using SplitsTree 4. Each set of parallel lines represents a split, in which the length is proportional to the weight of this split. Major splits are colored in different colors. (e and f) Visualization of SSNs using the Fruchterman–Reingold layout algorithm. Each node represents the RT protein of a retroelement, and each edge indicates protein-to-protein interaction weighted by 1/distance. The nodes are colored following the groups which they belong to. (g and h) SSNs with nodes colored based on the modularity class. (i and j) SSNs with nodes colored based on the CC.

Phylogenetic analysis of diverse retroelements shows that Kuafuorterviruses cluster with known RTVs, DIRS-like elements, and HEART retrotransposons with strong support (ultrafast bootstrap = 100%) (Fig. 1d; Supplementary Datasets S2 and S3; for convenience, this monophyletic group was termed as LTR-like retroelements), and Kuafuorterviruses do not fall within the diversity of any known LTR-like retroelement lineage. To further clarify the evolutionary relationships of Kuafuorterviruses and known LTR-like retroelements and exclude the confounding effect of the size of sampled sequences, we performed phylogenetic analyses using two representative RT protein datasets, namely, small (80 elements) and large (418 elements), with non-LTR retrotransposons as outgroups. For both datasets, maximum likelihood trees show that LTR-like retroelements grouped into nine distinct lineages and Kuafuorterviruses form clades that are only distantly related to the other LTR-like retroelements (Fig. 2a and b; Supplementary Datasets S4–7). It should be noted that the deep relationships among these lineages remain not well resolved with low to medium support values (Gladyshev and Arkhipova 2011). Moreover, we reconstructed phylogenetic networks (Huson and Bryant 2006) for both small and large RT protein datasets (Fig. 2c and d). While short rectilinear webbings at the center of the two networks indicate phylogenetic conflicts among distinct retroelement lineages possibly due to ancient recombination, the proximal splits are generally tree-like. The split of Kuafuorterviruses is clearly separated from the previously known LTR-like retroelements (Fig. 2c and d).

Moreover, we reconstructed SSNs to visualize sequence pairwise distances for both small and large RT protein datasets (Bastian et al. 2009). Proteins from the same retroelement taxonomy clustered together in the reconstructed SSNs (Fig. 2e and f). We used two algorithms, the MC and the CC statistic, to resolve cluster composition. For the small dataset, the MC and the CC algorithms divided retroelements into 10 and 9 clusters, respectively. The clusters agree well with the traditional taxonomy based on phylogenetic analyses (phylogenetic taxonomy) with the rand index of 0.767 for the MC algorithm vs. phylogenetic taxonomy and of 0.964 for the CC algorithm vs. phylogenetic taxonomy (Fig. 2e, g, and i). Both algorithms grouped Kuafuorterviruses as a single cluster, distinct from other LTR-like retroelements (Fig. 2e, g, and i). For the large dataset, clusters inferred from the MC algorithm agree better with phylogenetic taxonomy than the CC algorithm, with the rand index of 0.806 for the MC algorithm vs. phylogenetic taxonomy and of 0.335 for the CC algorithm vs. phylogenetic taxonomy (Fig. 2f, h, and j). The MC algorithm, but not the CC algorithm, recapitulated most of the canonical retroelement groups. The MC algorithm clustered Kuafuorterviruses as a single modularity class (Fig. 2f, h, and j). Taken together, phylogenetic analyses, phylogenetic networks, and SSNs strongly support that Kuafuorterviruses represent a novel superfamily of LTR retroelements. Together with the viral nature inferred from domain architectures, Kuafuorterviruses can be defined as a novel major RTV lineage, and we tentatively classified them as *Kuafuorterviridae*.

## Discussion

After decades of extensive analyses and discoveries of RTVs, eight families (*Metaviridae, Pseudoviridae, Belpaoviridae, Retroviridae, Caulimoviridae, Hepadnaviridae, Odinorterviridae*, and *Lokiorterviridae*) have been established. In this study, we systematically mined RTVs across eukaryote genomes. Surprisingly, we identified a novel lineage of RTVs, designated *Kuafuorterviridae*, in a wide range of animals. Although several Kuafuorterviruses (Troyka elements in *B. floridae* and *Nematostella vectensis*) were previously reported as retrotransposons in RepBase, they were grouped mistakenly into *Metaviridae* (Ty3/Gypsy superfamily) (Kapitonov and Jurka 2008). Evolutionary dynamics analyses suggest that Kuafuorterviruses are still actively proliferating in animals, reflecting the active transposable or mobile nature of Kuafuorterviruses, and the domain architecture supports the viral nature of Kuafuorterviruses. Additionally, phylogenetic analyses, phylogenetic networks, and SSNs strongly support that Kuafuorterviruses represent a novel lineage of RTVs, tentatively designated as *Kuafuorterviridae*. Therefore, we propose Kuafuorterviruses to be the ninth family of RTVs and the fifth superfamily of LTR retrotransposons. The discovery of Kuafuorterviruses refines our understanding of the diversity and evolution of RTVs and the classification system of LTR retrotransposons. Kuafuorterviruses have been largely neglected in previous animal genome sequencing projects. Therefore, Kuafuorterviruses should be implemented in an updated classification of RTVs and retrotransposons and routinely annotated in the future animal sequencing projects. Moreover, the discovery of Kuafuorterviruses also prompts our consideration that the diversity of RTVs and LTR retrotransposons is more complex than expected and there might be other neglected RTV lineages to be excavated.

## Supplementary Material

veae110_Supp

## Data Availability

All the data are available in the main text and supplementary materials.

## References

[R1] Ahlquist P Parallels among positive-strand RNA viruses, reverse-transcribing viruses and double-stranded RNA viruses. *Nat Rev Microbiol* 2006;4:371–82. doi: 10.1038/nrmicro1389 16582931 PMC7097367

[R2] Bastian M , Heymann S, Jacomy M Gephi: an open source software for exploring and manipulating networks. *Third Int AAAI Conf Weblogs Soc Media* 2009;3:361–62. doi: 10.1609/icwsm.v3i1.13937

[R3] Ellinghaus D , Kurtz S, Willhoeft U LTRharvest, an efficient and flexible software for de novo detection of LTR retrotransposons. *BMC Bioinf*. 2008;9:18. doi: 10.1186/1471-2105-9-18 PMC2253517 18194517

[R4] Gladyshev EA , Arkhipova IR A widespread class of reverse transcriptase-related cellular genes. *Proc Natl Acad Sci USA* 2011;108:20311–16. doi: 10.1073/pnas.1100266108 21876125 PMC3251080

[R5] Huson DH , Bryant D Application of phylogenetic networks in evolutionary studies. *Mol Biol Evol* 2006;23:254–67. doi: 10.1093/molbev/msj030 16221896

[R6] Kalyaanamoorthy S et al. ModelFinder: fast model selection for accurate phylogenetic estimates. *Nature Methods* 2017;14:587–89. doi: 10.1038/nmeth.4285 28481363 PMC5453245

[R7] Kapitonov VV , Jurka J Troyka – a distinctive group of Gypsy-like LTR retrotransposons inducing 3-bp target-site duplications. *Repbase Rep* 2008;8:510–16.

[R8] Katoh K , Standley DM MAFFT multiple sequence alignment software version 7: improvements in performance and usability. *Mol Biol Evol* 2013;30:772–80. doi: 10.1093/molbev/mst010 23329690 PMC3603318

[R9] Kelley LA , Sternberg MJ Protein structure prediction on the web: a case study using the phyre server. *Nat Protoc* 2009;4:363–71. doi: 10.1038/nprot.2009.2 19247286

[R10] Krupovic M et al. Ortervirales: new virus order unifying five families of reverse-transcribing viruses. *J Virol* 2018;92:e00515–18. doi: 10.1128/JVI.00515-18 29618642 PMC5974489

[R11] Munoz-Gomez SA et al. Nephromyces represents a diverse and novel lineage of the apicomplexa that has retained apicoplasts. *Genome Biol Evol* 2019;11:2727–40. doi: 10.1093/gbe/evz155 31328784 PMC6777426

[R12] Nguyen LT et al. IQ-TREE: a fast and effective stochastic algorithm for estimating maximum-likelihood phylogenies. *Mol Biol Evol* 2015;32:268–74. doi: 10.1093/molbev/msu300 25371430 PMC4271533

[R13] Price MN , Dehal PS, Arkin AP FastTree 2—approximately maximum-likelihood trees for large alignments. *PLoS One* 2010;5:e9490. doi: 10.1371/journal.pone.0009490 PMC2835736 20224823

[R14] Sharma PL , Nurpeisov V, Schinazi RF Retrovirus reverse transcriptases containing a modified YXDD motif. *Antiviral Chem Chemother* 2005;16:169–82. doi: 10.1177/095632020501600303 16004080

[R15] T. HD et al. UFBoot2: improving the ultrafast bootstrap approximation. *Mol Biol Evol* 2018;35:518–22. doi: 10.1093/molbev/msx281 29077904 PMC5850222

[R16] Telford MJ , Budd GE, Philippe H Phylogenomic insights into animal evolution. *Curr Biol* 2015;25:R876–87. doi: 10.1016/j.cub.2015.07.060 26439351

[R17] Walker PJ et al. Changes to virus taxonomy and the statutes ratified by the international committee on taxonomy of viruses (2020). *Arch. Virol* 2020;165:2737–48. doi: 10.1007/s00705-020-04752-x 32816125

[R18] Wang JH , Han GZ A sister lineage of sampled retroviruses corroborates the complex evolution of retroviruses. *Mol Biol Evol* 2021;38:1031–39. doi: 10.1093/molbev/msaa272 33249491 PMC7947760

[R19] Wang JH , Han GZ A missing link between retrotransposons and retroviruses. *mBio* 2022;13:e0018722. doi: 10.1128/mbio.00187-22 PMC9040795 35289644

[R20] Wicker T et al. A unified classification system for eukaryotic transposable elements. *Nat Rev Genet* 2007;8:973–82. doi: 10.1038/nrg2165 17984973

[R21] Y. L et al. Rooting the animal tree of life. *Mol Biol Evol* 2021;38:4322–33. doi: 10.1093/molbev/msab170 34097041 PMC8476155

[R22] Zimmermann L et al. A completely reimplemented MPI bioinformatics toolkit with a new hhpred server at its core. *J Mol Biol* 2018;430:2237–43. doi: 10.1016/j.jmb.2017.12.007 29258817

