# Supplementary material for: Kuafuorterviruses, a novel major lineage of reverse-transcribing viruses
Source: Virus Evol. 2024 Dec 19;10(1):veae110. doi: 10.1093/ve/veae110 (PMC11673190; doi:10.1093/ve/veae110)
Supplement: veae110_Supp [file veae110_supp.zip › suppl_data/Supplementary Material.pdf]

Supplementary Material for

Kuafuorterviruses, a novel major lineage of reverse-transcribing viruses

Supplementary Table S1.
Information of the genomes where Kuafuorterviruses were identified.

Group	Phylum	Species	Accession No.
Animals	Annelida	*Lamellibrachia luymesi*	GCA_009193005.1
	Arthropoda	*Acartia tonsa*	GCA_900241095.1
	Arthropoda	*Amphibalanus amphitrite*	GCA_009805615.1
	Arthropoda	*Drosophila ficusphila*	GCA_000220665.2
	Arthropoda	*Gammarus roeselii*	GCA_016164225.1
	Arthropoda	*Macrobrachium nipponense*	GCA_015104395.1
	Arthropoda	*Palaemon carinicauda*	GCA_004011675.1
	Arthropoda	*Pandalus platyceros*	GCA_005815305.1
	Arthropoda	*Semibalanus balanoides*	GCA_014673585.1
	Cephalochordata	*Branchiostoma floridae*	GCA_013266295.2
	Cephalochordata	*Branchiostoma lanceolatum*	GCA_900088365.1
	Cnidaria	*Acropora acuminata*	GCA_014633975.1
	Cnidaria	*Acropora awi*	GCA_014634005.1
	Cnidaria	*Acropora cytherea*	GCA_014634045.1
	Cnidaria	*Acropora echinata*	GCA_014634105.1
	Cnidaria	*Acropora florida*	GCA_014634605.1
	Cnidaria	*Acropora gemmifera*	GCA_014634125.1
	Cnidaria	*Acropora hyacinthus*	GCA_014634145.1
	Cnidaria	*Acropora intermedia*	GCA_014634585.1

Cnidaria	*Acropora microphthalma*	GCA_014634165.1
Cnidaria	*Acropora muricata*	GCA_014634545.1
Cnidaria	*Acropora nasuta*	GCA_014634205.1
Cnidaria	*Acropora selago*	GCA_014634525.1
Cnidaria	*Acropora tenuis*	GCA_014633955.1
Cnidaria	*Acropora yongei*	GCA_014634225.1
Cnidaria	*Actinia equina*	GCA_011057435.1
Cnidaria	*Anemonia viridis*	GCA_900234385.1
Cnidaria	*Astreopora myriophthalma*	GCA_014634185.1
Cnidaria	*Calvadosia cruxmelitensis*	GCA_900245855.1
Cnidaria	*Cassiopea xamachana*	GCA_900291935.1
Cnidaria	*Clytia hemisphaerica*	GCA_902728285.1
Cnidaria	*Heteractis crispa*	GCA_015164035.1
Cnidaria	*Heteractis magnifica*	GCA_011763375.1
Cnidaria	*Montipora cactus*	GCA_014634245.1
Cnidaria	*Montipora capitata*	GCA_006542545.1
Cnidaria	*Montipora efflorescens*	GCA_014634505.1
Cnidaria	*Morbakka virulenta*	GCA_003991215.1
Cnidaria	*Nemopilema nomurai*	GCA_003864495.1
Cnidaria	*Pachyseris speciosa*	GCA_016490735.1
Cnidaria	*Phymanthus crucifer*	GCA_009858155.1
Cnidaria	*Pocillopora verrucosa*	GCA_014529365.1
Cnidaria	*Porites rus*	GCA_900290455.1
Cnidaria	*Renilla reniformis*	GCA_900177555.1
Cnidaria	*Sanderia malayensis*	GCA_013076295.1

Cnidaria	*Stichodactyla helianthus*	GCA_015163945.1
Cnidaria	*Stichodactyla mertensii*	GCA_011800005.1
Cnidaria	*Trachythela* sp. YZ-2020	GCA_016169945.1
Echinodermata	*Actinopyga echinites*	GCA_010015985.1
Echinodermata	*Australostichopus mollis*	GCA_900067635.1
Echinodermata	*Eucidaris tribuloides*	GCA_001188425.1
Echinodermata	*Holothuria glaberrima*	GCA_009936505.1
Echinodermata	*Lytechinus pictus*	GCA_015342785.1
Echinodermata	*Ophionereis fasciata*	GCA_900067615.1
Echinodermata	*Ophiothrix spiculata*	GCA_000969725.1
Echinodermata	*Patiriella regularis*	GCA_900067625.1
Echinodermata	*Pisaster ochraceus*	GCA_010994315.1
Echinodermata	*Stichopus horrens*	GCA_009801055.1
Hemichordata	*Ptychodera flava*	GCA_001465055.1
Mollusca	*Acanthopleura granulata*	GCA_016165875.1
Mollusca	*Architeuthis dux*	GCA_006491835.1
Mollusca	*Archivesica marissinica*	GCA_014843695.1
Mollusca	*Argopecten irradians irradians*	GCA_004382745.1
Mollusca	*Bathymodiolus platifrons*	GCA_002080005.1
Mollusca	*Chrysomallon squamiferum*	GCA_012295275.1
Mollusca	*Crassostrea hongkongensis*	GCA_015776775.1
Mollusca	*Cyclina sinensis*	GCA_012932295.1
Mollusca	*Dracogyra subfuscus*	GCA_016106625.1
Mollusca	*Dreissena rostriformis*	GCA_007657795.1
Mollusca	*Euprymna scolopes*	GCA_004765925.1

Mollusca	*Gigantopelta aegis*	GCA_016097555.1
Mollusca	*Haliotis laevigata*	GCA_008038995.1
Mollusca	*Haliotis rubra*	GCA_003918875.1
Mollusca	*Haliotis rufescens*	GCA_003343065.1
Mollusca	*Limacina bulimoides*	GCA_009866985.1
Mollusca	*Limnoperna fortunei*	GCA_003130415.1
Mollusca	*Lutraria rhynchaena*	GCA_008271625.1
Mollusca	*Magallana hongkongensis*	GCA_016163765.1
Mollusca	*Mercenaria mercenaria*	GCA_014805675.1
Mollusca	*Modiolus philippinarum*	GCA_002080025.1
Mollusca	*Mytilus coruscus*	GCA_017311375.1
Mollusca	*Mytilus galloprovincialis*	GCA_900618805.1
Mollusca	*Ostrea lurida*	GCA_903981925.1
Mollusca	*Panopea generosa*	GCA_902825435.1
Mollusca	*Pinctada imbricata*	GCA_002216045.1
Mollusca	*Pinna nobilis*	GCA_016161895.1
Mollusca	*Ruditapes philippinarum*	GCA_009026015.1
Mollusca	*Saccostrea glomerata*	GCA_003671525.1
Mollusca	*Sepia pharaonis*	GCA_903632075.3
Mollusca	*Sinonovacula constricta*	GCA_007844125.1
Mollusca	*Tegillarca granosa*	GCA_013375625.1
Mollusca	*Watasenia scintillans*	GCA_015471945.1
Nemertea	*Notospermus geniculatus*	GCA_002633025.1
Phoronida	*Phoronis australis*	GCA_002633005.1
Tunicata	*Halocynthia aurantium*	GCA_013436065.1

Tunicata	*Halocynthia roretzi*	GCA_013436055.1
Vertebrata	*Anabarilius grahami*	GCA_003731715.1
Vertebrata	*Anguilla anguilla*	GCA_013347855.1
Vertebrata	*Anguilla japonica*	GCA_003597225.1
Vertebrata	*Anguilla marmorata*	GCA_901111315.1
Vertebrata	*Anguilla megastoma*	GCA_901111305.1
Vertebrata	*Anguilla obscura*	GCA_901111295.1
Vertebrata	*Anguilla rostrata*	GCA_001606085.1
Vertebrata	*Astyanax mexicanus*	GCA_000372685.2
Vertebrata	*Benthosema glaciale*	GCA_900323375.1
Vertebrata	*Borostomias antarcticus*	GCA_900323325.1
Vertebrata	*Bregmaceros cantori*	GCA_900302395.1
Vertebrata	*Carapus acus*	GCA_900312935.1
Vertebrata	*Carassius auratus*	GCA_003368295.1
Vertebrata	*Chanos chanos*	GCA_902362185.1
Vertebrata	*Chiloscyllium plagiosum*	GCA_004010195.1
Vertebrata	*Chiloscyllium punctatum*	GCA_003427335.1
Vertebrata	*Cirrhinus molitorella*	GCA_004028445.1
Vertebrata	*Clupea harengus*	GCA_900700415.1
Vertebrata	*Coilia nasus*	GCA_007927625.1
Vertebrata	*Cromileptes altivelis*	GCA_013133815.1
Vertebrata	*Culter alburnus*	GCA_009869775.1
Vertebrata	*Cyprinus carpio*	GCA_000951615.2
Vertebrata	*Danio tinwini*	GCA_903798205.1
Vertebrata	*Diplodus sargus*	GCA_903131615.1

Vertebrata	*Diretmoides pauciradiatus*	GCA_900660315.1
Vertebrata	*Diretmus argenteus*	GCA_900660295.1
Vertebrata	*Entelurus aequoreus*	GCA_901007785.1
Vertebrata	*Epinephelus coioides*	GCA_900536245.1
Vertebrata	*Epinephelus fuscoguttatus*	GCA_011397635.1
Vertebrata	*Epinephelus lanceolatus*	GCA_005281545.1
Vertebrata	*Epinephelus moara*	GCA_006386435.1
Vertebrata	*Eptatretus burgeri*	GCA_900186335.2
Vertebrata	*Guaruba guarouba*	GCA_011800095.1
Vertebrata	*Hoplostethus atlanticus*	GCA_900660355.1
Vertebrata	*Hypophthalmichthys molitrix*	GCA_004764525.1
Vertebrata	*Hypophthalmichthys nobilis*	GCA_004193235.1
Vertebrata	*Labeo catla*	GCA_014525385.1
Vertebrata	*Labeo gonius*	GCA_013461565.1
Vertebrata	*Lampris guttatus*	GCA_900302545.1
Vertebrata	*Leuciscus waleckii*	GCA_900092035.1
Vertebrata	*Mallotus villosus*	GCA_903064625.1
Vertebrata	*Megalobrama amblycephala*	GCA_009869865.1
Vertebrata	*Megalops cyprinoides*	GCA_013368585.1
Vertebrata	*Merluccius capensis*	GCA_900312945.1
Vertebrata	*Merluccius merluccius*	GCA_900312545.1
Vertebrata	*Merluccius polli*	GCA_900312625.1
Vertebrata	*Nerophis ophidion*	GCA_901007905.1
Vertebrata	*Odontesthes bonariensis*	GCA_014825785.1
Vertebrata	*Onychostoma macrolepis*	GCA_012432095.1

Vertebrata	*Oxygymnocypris stewartii*	GCA_003573665.1
Vertebrata	*Pachypanchax playfairii*	GCA_006937955.1
Vertebrata	*Pagrus major*	GCA_002897255.1
Vertebrata	*Parasudis fraserbrunneri*	GCA_900302295.1
Vertebrata	*Pimephales promelas*	GCA_016745375.1
Vertebrata	*Poropuntius huangchuchieni*	GCA_004124795.1
Vertebrata	*Psittacus timneh*	GCA_009867315.1
Vertebrata	*Regalecus glesne*	GCA_900302585.1
Vertebrata	*Rhincodon typus*	GCA_001642345.2
Vertebrata	*Salarias fasciatus*	GCA_902148845.1
Vertebrata	*Sardina pilchardus*	GCA_900499035.1
Vertebrata	*Scyliorhinus canicula*	GCA_902713615.1
Vertebrata	*Scyliorhinus torazame*	GCA_003427355.1
Vertebrata	*Selene dorsalis*	GCA_900303245.1
Vertebrata	*Sinocyclocheilus anshuiensis*	GCA_001515605.1
Vertebrata	*Sinocyclocheilus grahami*	GCA_001515645.1
Vertebrata	*Sinocyclocheilus rhinocerous*	GCA_001515625.1
Vertebrata	*Sparus aurata*	GCA_900880675.1
Vertebrata	*Stylephorus chordatus*	GCA_900312615.1
Vertebrata	*Tenualosa ilisha*	GCA_015244755.2
Vertebrata	*Thamnaconus septentrionalis*	GCA_009823395.1
Vertebrata	*Trachurus trachurus*	GCA_905171665.1
Vertebrata	*Tragelaphus imberbis*	GCA_006410775.1
Vertebrata	*Triplophysa siluroides*	GCA_006030095.1
Vertebrata	*Trisopterus minutus*	GCA_900302415.1

Protists	Apicomplexa	*Nephromyces* sp. ex *Molgula occidentalis*	GCA_004523865.1

Supplementary Table S2.
Copy numbers of Kuafuorterviruses in species.

Taxonomy/Species	Copy No. of Kuafuorterviruses with LTRs
**Annelida**	**11**
Lamellibrachia luymesi	11
**Arthropoda**	**20**
Acartia tonsa	8
Amphibalanus amphitrite	11
Semibalanus balanoides	1
**Cephalochordata**	**9**
Branchiostoma floridae	7
Branchiostoma lanceolatum	2
**Cnidaria**	**580**
Acropora acuminata	12
Acropora awi	10
Acropora cytherea	14
Acropora echinata	16
Acropora florida	17
Acropora gemmifera	13
Acropora hyacinthus	8
Acropora intermedia	12
Acropora microphthalma	10
Acropora muricata	16
Acropora nasuta	7
Acropora selago	13
Acropora tenuis	12
Acropora yongei	35
Actinia equina	23
Astreopora myriophthalma	18
Cassiopea xamachana	1
Clytia hemisphaerica	31
Heteractis crispa	1
Montipora cactus	28
Montipora capitata	15
Montipora efflorescens	23
Morbakka virulenta	46
Nemopilema nomurai	2
Pachyseris speciosa	143
Pocillopora verrucosa	1
Porites rus	1

Sanderia malayensis	1
Trachythela sp. YZ-2020	51
**Echinodermata**	**29**
Lytechinus pictus	29
**Mollusca**	**349**
Architeuthis dux	3
Argopecten irradians irradians	25
Crassostrea hongkongensis	12
Cyclina sinensis	5
Dracogyra subfuscus	7
Dreissena rostriformis	1
Euprymna scolopes	9
Gigantopelta aegis	48
Haliotis laevigata	10
Haliotis rubra	29
Haliotis rufescens	56
Limacina bulimoides	1
Limnoperna fortunei	3
Lutraria rhynchaena	1
Magallana hongkongensis	12
Mercenaria mercenaria	16
Mytilus coruscus	19
Mytilus galloprovincialis	20
Ostrea lurida	4
Panopea generosa	15
Ruditapes philippinarum	3
Saccostrea glomerata	12
Sinonovacula constricta	20
Tegillarca granosa	18
**Nemertea**	**6**
Notospermus geniculatus	6
**Phoronida**	**16**
Phoronis australis	16
**Tunicata**	**4**
Halocynthia aurantium	1
Halocynthia roretzi	3
**Vertebrata**	**76**
Anabarilius grahami	3
Anguilla obscura	1
Anguilla rostrata	1
Astyanax mexicanus	3

Benthosema glaciale	1
Bregmaceros cantori	2
Carassius auratus	1
Clupea harengus	6
Coilia nasus	3
Culter alburnus	1
Cyprinus carpio	1
Diplodus sargus	1
Diretmoides pauciradiatus	1
Epinephelus coioides	1
Epinephelus fuscoguttatus	2
Epinephelus lanceolatus	1
Epinephelus moara	3
Hypophthalmichthys nobilis	1
Labeo gonius	1
Leuciscus waleckii	1
Megalobrama amblycephala	2
Odontesthes bonariensis	3
Onychostoma macrolepis	1
Oxygymnocypris stewartii	9
Salarias fasciatus	1
Sardina pilchardus	3
Scyliorhinus canicula	4
Selene dorsalis	1
Sinocyclocheilus anshuiensis	3
Sinocyclocheilus grahami	5
Sinocyclocheilus rhinocerous	2
Sparus aurata	1
Tenualosa ilisha	4
Trachurus trachurus	2

Supplementary Table S3.

Length and domain architectures of Kuafuorterviruses with LTRs.

Taxonomy	Species	Position	Length	Domain architecture
Annelida	*Lamellibrachia luymesi*	SDWI01000042.1:976620_984977	8357	MGDG_synth,Exo_endo_phos_2,RVT_1
		SDWI01000238.1:389598_395224	5626	Asp_protease_2,RVT_1,RT_RNaseH_2
		SDWI01000260.1:68082_73620	5538	Asp_protease_2,RVT_1,RT_RNaseH_2
		SDWI01000405.1:158573_171447	12874	RVT_1,RT_RNaseH_2,Integrase_H2C2
		SDWI01000517.1:192397_197380	4983	Asp_protease_2,RVT_1,RT_RNaseH_2,Integrase_H2C2
		SDWI01000545.1:120188_133651	13463	RVT_1
		SDWI01000691.1:37336_42864	5528	Asp_protease_2,RVT_1,RT_RNaseH_2
		SDWI01001354.1:5369_11525	6156	Asp_protease_2,RVT_1,RT_RNaseH_2
		SDWI01002039.1:35664_40978	5314	Asp_protease_2,RT_RNaseH
		SDWI01002506.1:1217_6617	5400	Asp_protease_2,RVT_1,RT_RNaseH_2
		SDWI01003266.1:9994_15538	5544	Asp_protease_2,RVT_1,RT_RNaseH_2
Arthropoda	*Acartia tonsa*	LS050600.1:6506_13032	6526	RVT_1,RT_RNaseH_2
		LS043258.1:1164_7524	6360	RT_RNaseH_2,Integrase_H2C2
		LS043037.1:10521_16809	6288	Asp_protease_2,RVT_1,RT_RNaseH_2,Integrase_H2C2
		LS044436.1:8628_15025	6397	Asp_protease_2,RVT_1,RVT_1,RT_RNaseH_2
		LS060320.1:15_6892	6877	gag-asp_proteas,RVT_1,RT_RNaseH_2,Integrase_H2C2
		LS066968.1:5_6979	6974	gag-asp_proteas,RVT_1,RT_RNaseH_2,Integrase_H2C2
		LS065053.1:824_7406	6582	RVT_1,RT_RNaseH_2,Integrase_H2C2,rve
		LS070959.1:13_6327	6314	Asp_protease_2,RVT_1,RVT_1,RT_RNaseH_2,Integrase_H2C2
	Amphibalanus amphitrite	VIIS01000039.1:274942_280661	5719	RVT_1,RT_RNaseH_2,Integrase_H2C2,rve
		VIIS01000139.1:22640_28470	5830	RVT_1,RT_RNaseH_2,Integrase_H2C2,rve
		VIIS01000459.1:117163_127417	10254	RVT_1,RT_RNaseH_2,SLATT_3,RVT_1
		VIIS01000475.1:37772_43382	5610	gag-asp_proteas,RVT_1,RT_RNaseH_2,Integrase_H2C2
		VIIS01000802.1:197274_205622	8348	gag-asp_proteas,RVT_1,RVT_1,RVT_1,RVT_1,RT_RNaseH_2,Integrase_H2C2
		VIIS01000876.1:117564_123180	5616	gag-asp_proteas,RVT_1,RT_RNaseH_2,Integrase_H2C2
		VIIS01001423.1:10333_17495	7162	gag-asp_proteas,RVT_1,RT_RNaseH_2,Integrase_H2C2
		VIIS01001520.1:25778_31631	5853	RVT_1,RT_RNaseH_2,Integrase_H2C2,rve
		VIIS01001636.1:233739_238329	4590	RVT_1,RT_RNaseH_2,Integrase_H2C2,rve
		VIIS01001985.1:365564_370834	5270	gag-asp_proteas,RVT_1,RT_RNaseH,Integrase_H2C2
		VIIS01001992.1:367271_372784	5513	gag-asp_proteas,RVT_1,RT_RNaseH_2,Integrase_H2C2
	Semibalanus balanoides	VOPJ01002082.1:1181_11771	10590	RVT_1,RT_RNaseH_2,gag-asp_proteas
Cephalochor	*Branchiostoma*	CM023824.1:10545386_10551159	5773	gag-asp_proteas,RVT_1,RT_RNaseH_2,Integrase_H2C2

data	*floridae*	CM023824.1:24271032_24276796	5764	gag-asp_proteas,RVT_1,RT_RNaseH_2,Integrase_H2C2
		CM023826.1:19481586_19486876	5290	Asp_protease_2,RVT_1,RT_RNaseH_2,Integrase_H2C2
		CM023827.1:9349443_9355217	5774	gag-asp_proteas,RVT_1,RT_RNaseH_2,Integrase_H2C2
		CM023828.1:18388086_18393728	5642	Asp_protease_2,RVT_1,RT_RNaseH_2,Integrase_H2C2
		CM023830.1:1364546_1370400	5854	gag-asp_proteas,RVT_1,RT_RNaseH_2,Integrase_H2C2
		CM023831.1:17462481_17468449	5968	gag-asp_proteas,RT_RNaseH_2,Integrase_H2C2
	Branchiostoma lanceolatum	FLLO01000013.1:3905593_3911699	6106	gag-asp_proteas,RVT_1,RT_RNaseH_2,Integrase_H2C2
		FLLO01001127.1:4901_11234	6333	gag-asp_proteas,RVT_1,RT_RNaseH_2,Integrase_H2C2
Cnidaria	*Acropora acuminata*	BLEZ01000138.1:19720_25321	5601	Asp_protease_2,RVT_1,RT_RNaseH_2,Integrase_H2C2
		BLEZ01000157.1:67738_73767	6029	Asp_protease_2,RT_RNaseH,Integrase_H2C2
		BLEZ01001243.1:206597_212095	5498	Asp_protease_2,RVT_1,RT_RNaseH_2,rve_3
		BLEZ01001456.1:41614_46602	4988	Asp_protease_2,RVT_1,RT_RNaseH_2,RT_RNaseH,Integrase_H2C2
		BLEZ01001660.1:29936_35451	5515	Asp_protease_2,RVT_1,RVT_1,RT_RNaseH
		BLEZ01001848.1:5806_10931	5125	RVT_1,Integrase_H2C2
		BLEZ01002001.1:152051_157646	5595	RVT_1,RT_RNaseH_2
		BLEZ01002205.1:742449_747978	5529	gag-asp_proteas,RVT_1,Integrase_H2C2
		BLEZ01002205.1:1007094_1012951	5857	Asp_protease_2,RVT_1,RT_RNaseH_2,Integrase_H2C2
		BLEZ01002291.1:123572_133082	9510	DUF4164
		BLEZ01002708.1:610942_619434	8492	gag-asp_proteas,RVT_1,RT_RNaseH_2,Integrase_H2C2
		BLEZ01002809.1:520952_526533	5581	Asp_protease_2,RT_RNaseH_2
	Acropora awi	BLFA01000355.1:54671_61109	6438	Asp_protease_2,RVT_1,RT_RNaseH_2,Integrase_H2C2
		BLFA01000511.1:592669_600106	7437	RVT_1,RT_RNaseH_2,Integrase_H2C2
		BLFA01000867.1:1661461_1667006	5545	Asp_protease_2,RVT_1,RT_RNaseH_2,Integrase_H2C2
		BLFA01001482.1:276304_280636	4332	
		BLFA01001496.1:914040_919394	5354	Asp_protease_2,RVT_1,RT_RNaseH,Integrase_H2C2,rve_3
		BLFA01001691.1:111437_116213	4776	RT_RNaseH,Integrase_H2C2
		BLFA01002089.1:240192_245463	5271	DUF5716,RT_RNaseH_2,Integrase_H2C2
		BLFA01002483.1:106854_121792	14938	RVT_1,RT_RNaseH_2,Integrase_H2C2
		BLFA01002578.1:204373_209826	5453	Asp_protease_2,RVT_1
		BLFA01002578.1:263521_269680	6159	Asp_protease_2,RVT_1,RVT_1,RT_RNaseH_2,Integrase_H2C2
	Acropora cytherea	BLFB01000111.1:1060028_1065712	5684	Asp_protease_2,RVT_1,RT_RNaseH_2,Integrase_H2C2

	BLFB01000111.1:1176977_1182563	5586	Asp_protease_2,RVT_1,RT_RNaseH_2,Integrase_H2C2
	BLFB01000171.1:484549_489634	5085	gag-asp_proteas,RVT_1,RT_RNaseH,Integrase_H2C2
	BLFB01000184.1:1020028_1026130	6102	gag-asp_proteas,RVT_1,RT_RNaseH_2,Integrase_H2C2
	BLFB01000613.1:507977_513592	5615	RVT_1,RT_RNaseH_2,Integrase_H2C2
	BLFB01001373.1:1671397_1676915	5518	Asp_protease_2,RVT_1,RT_RNaseH_2,Integrase_H2C2
	BLFB01001526.1:19605_25088	5483	gag-asp_proteas,RVT_1,RT_RNaseH_2
	BLFB01001861.1:531783_537614	5831	gag-asp_proteas,RVT_1,RT_RNaseH_2,Integrase_H2C2
	BLFB01002320.1:2945604_2955177	9573	RT_RNaseH_2,Integrase_H2C2
	BLFB01002365.1:730843_736267	5424	Asp_protease_2,RT_RNaseH_2,Integrase_H2C2
	BLFB01002422.1:180179_185733	5554	gag-asp_proteas,RVT_1,RT_RNaseH_2,Integrase_H2C2
	BLFB01002648.1:491703_497215	5512	RVT_1,Integrase_H2C2
	BLFB01003136.1:82157_87696	5539	Asp_protease_2,RT_RNaseH_2,Integrase_H2C2
	BLFB01003744.1:3659668_3664956	5288	GIY-YIG
Acropora echinata	BLFD01000254.1:528965_535432	6467	Asp_protease_2,RVT_1
	BLFD01000319.1:115701_121313	5612	Asp_protease_2,RVT_1,RT_RNaseH_2,Integrase_H2C2
	BLFD01000440.1:479301_484259	4958	RT_RNaseH_2
	BLFD01000877.1:1716760_1721858	5098	DUF4164,Asp_protease_2,RVT_1,RT_RNaseH_2,Integrase_H2C2
	BLFD01000913.1:148073_152570	4497	RT_RNaseH_2
	BLFD01000967.1:63056_68613	5557	gag-asp_proteas,RT_RNaseH,Integrase_H2C2
	BLFD01001013.1:1290486_1296013	5527	Asp_protease_2,RVT_1,RT_RNaseH_2,Integrase_H2C2,rve_3
	BLFD01001061.1:112898_115877	2979	gag-asp_proteas,RVT_1
	BLFD01001068.1:672974_678471	5497	gag-asp_proteas,RVT_1,RT_RNaseH_2,Integrase_H2C2
	BLFD01001221.1:1526495_1529746	3251	
	BLFD01001585.1:18561_24126	5565	Asp_protease_2,RVT_1,RT_RNaseH_2,Integrase_H2C2
	BLFD01001662.1:2148064_2153670	5606	RT_RNaseH_2
	BLFD01001706.1:861170_866692	5522	RVT_1,RT_RNaseH
	BLFD01001749.1:43488_49069	5581	Asp_protease_2,RVT_1,RVT_1,RT_RNaseH_2
	BLFD01001838.1:319094_323115	4021	gag-asp_proteas
	BLFD01001906.1:606362_611398	5036	gag-asp_proteas,RVT_1,RT_RNaseH_2
Acropora	BLFE01000865.1:643263_648193	4930	gag-asp_proteas,RT_RNaseH,Integrase_H2C2

florida	BLFE01001298.1:33820_39428	5608	Asp_protease_2,RVT_1,RT_RNaseH_2,Integrase_H2C2
	BLFE01001911.1:40540_46097	5557	Asp_protease_2,RVT_1,RT_RNaseH_2,Integrase_H2C2
	BLFE01002153.1:354329_358672	4343	
	BLFE01002165.1:237573_244746	7173	Asp_protease_2,RVT_1,RT_RNaseH_2,Integrase_H2C2
	BLFE01002380.1:7507_13751	6244	Asp_protease_2,RVT_1,RT_RNaseH_2,Integrase_H2C2
	BLFE01002764.1:75269_80755	5486	Asp_protease_2,RVT_1,RT_RNaseH_2,RT_RNaseH,rve_3
	BLFE01003257.1:87747_93323	5576	RVT_1,RT_RNaseH_2
	BLFE01003340.1:524335_538108	13773	zf-C2H2_2
	BLFE01003759.1:4008_9579	5571	RT_RNaseH_2,Integrase_H2C2
	BLFE01004747.1:92244_101000	8756	RVT_1,Asp_protease_2,RT_RNaseH,rve
	BLFE01004798.1:95137_100568	5431	RVT_1,RVT_1,RT_RNaseH_2,Integrase_H2C2
	BLFE01004798.1:146352_151831	5479	Asp_protease_2,RVT_1,RT_RNaseH_2,Integrase_H2C2
	BLFE01004804.1:127126_130069	2943	RVT_1,RT_RNaseH
	BLFE01005083.1:278734_284361	5627	Asp_protease_2,RVT_1,RT_RNaseH_2,Integrase_H2C2
	BLFE01005497.1:42870_47664	4794	RVT_1,RT_RNaseH_2,Integrase_H2C2
	BLFE01006047.1:587794_592907	5113	RT_RNaseH_2,Integrase_H2C2
Acropora gemmifera	BLFF01000050.1:561184_566780	5596	Asp_protease_2,RVT_1,RT_RNaseH,Integrase_H2C2
	BLFF01000050.1:622371_631801	9430	gag-asp_proteas,RVT_1,RT_RNaseH_2,Integrase_H2C2
	BLFF01000120.1:16114_21042	4928	gag-asp_proteas,RVT_1,RT_RNaseH_2,Integrase_H2C2
	BLFF01000222.1:21517_32374	10857	Asp_protease_2,RVT_1,RT_RNaseH_2,Integrase_H2C2,rve_3
	BLFF01000277.1:131949_137521	5572	RT_RNaseH_2,Integrase_H2C2
	BLFF01000386.1:88339_95484	7145	RT_RNaseH_2
	BLFF01000460.1:10_2753	2743	Pkinase,Ribonuc_2-5A
	BLFF01000542.1:534010_538894	4884	RVT_1,RT_RNaseH_2,Integrase_H2C2
	BLFF01000851.1:687637_693221	5584	RVT_1,RT_RNaseH,Integrase_H2C2
	BLFF01001064.1:538219_543744	5525	gag-asp_proteas,RT_RNaseH,Integrase_H2C2,Integrase_H2C2
	BLFF01001298.1:13274_16917	3643	Asp_protease_2
	BLFF01001650.1:1762732_1767138	4406	zf-RVT,Integrase_H2C2,rve_3
	BLFF01001824.1:688572_695301	6729	RVT_1,RVT_1,RT_RNaseH_2,Integrase_H2C2
Acropora hyacinthus	BLFG01000085.1:227620_233028	5408	gag-asp_proteas,RT_RNaseH,Integrase_H2C2
	BLFG01000316.1:1068761_1077651	8890	gag-asp_proteas,RVT_1,RT_RNaseH_2
	BLFG01000499.1:808682_814190	5508	gag-asp_proteas,RVT_1,RT_RNaseH_2,Integrase_H2C2
	BLFG01001557.1:411867_416445	4578	gag-asp_proteas,RVT_1,RT_RNaseH_2
	BLFG01001589.1:2334875_23393	4429	gag-asp_proteas,RT_RNaseH

	04		
	BLFG01001670.1:824846_830438	5592	Asp_protease_2,RT_RNaseH_2,Integrase_H2C2
	BLFG01001797.1:17376_22999	5623	Asp_protease_2,RT_RNaseH_2,Integrase_H2C2
	BLFG01001939.1:417803_424276	6473	TetR_C_24,Asp_protease_2,TetR_C_24,RVT_1,RT_RNaseH,Integrase_H2C2
Acropora intermedia	BLFH01000728.1:513966_519131	5165	gag-asp_proteas,RT_RNaseH_2,Integrase_H2C2
	BLFH01000817.1:592425_597792	5367	Asp_protease_2,RVT_1,RT_RNaseH,rve_3
	BLFH01000980.1:2429_7363	4934	RT_RNaseH,Integrase_H2C2
	BLFH01001120.1:518143_523714	5571	gag-asp_proteas,RVT_1,RT_RNaseH,Integrase_H2C2
	BLFH01001834.1:32270_36614	4344	
	BLFH01002220.1:147206_160543	13337	Asp_protease_2,RVT_1,RT_RNaseH_2
	BLFH01002486.1:303124_308231	5107	RT_RNaseH_2,Integrase_H2C2
	BLFH01003940.1:310588_316128	5540	gag-asp_proteas,RT_RNaseH_2,Integrase_H2C2
	BLFH01004208.1:124690_137395	12705	Neur
	BLFH01004288.1:449176_457925	8749	Asp_protease_2,RT_RNaseH_2,Integrase_H2C2
	BLFH01004672.1:90355_96078	5723	Asp_protease_2,RVT_1,RT_RNaseH_2,Integrase_H2C2,rve_3
	BLFH01005632.1:205039_209816	4777	RT_RNaseH,Integrase_H2C2
Acropora microphthalma	BLFI01000603.1:135029_140614	5585	Asp_protease_2,RVT_1,RT_RNaseH_2,Integrase_H2C2
	BLFI01001336.1:67620_73415	5795	gag-asp_proteas,RT_RNaseH_2,Integrase_H2C2
	BLFI01001343.1:1007068_1012620	5552	gag-asp_proteas,RVT_1,RT_RNaseH_2,Integrase_H2C2
	BLFI01001408.1:24495_29625	5130	Asp_protease_2,RT_RNaseH_2,Integrase_H2C2,rve
	BLFI01002415.1:386815_391182	4367	RVT_1,RT_RNaseH_2,Integrase_H2C2
	BLFI01002739.1:323156_328707	5551	RVT_1,RT_RNaseH_2,Integrase_H2C2
	BLFI01003632.1:578714_583536	4822	RVT_1,RT_RNaseH_2,Integrase_H2C2
	BLFI01003932.1:424126_429698	5572	RVT_1,RT_RNaseH_2
	BLFI01004454.1:105736_110894	5158	RVT_1,Integrase_H2C2
	BLFI01004757.1:303014_307488	4474	gag-asp_proteas,RT_RNaseH
Acropora muricata	BLFJ01000198.1:66181_71592	5411	Asp_protease_2,RT_RNaseH,Integrase_H2C2,rve_3
	BLFJ01000797.1:11466_17023	5557	RVT_1,RT_RNaseH_2,Integrase_H2C2
	BLFJ01000867.1:47545_59555	12010	Asp_protease_2,RVT_1,RT_RNaseH_2
	BLFJ01002018.1:56601_61731	5130	Asp_protease_2,RVT_1,RT_RNaseH_2,RT_RNaseH,Integrase_H2C2
	BLFJ01002118.1:4329_15945	11616	Asp_protease_2,RVT_1,RT_RNaseH_2,Integrase_H2C2
	BLFJ01002482.1:1089955_1095163	5208	gag-asp_proteas,Integrase_H2C2
	BLFJ01002591.1:20133_25688	5555	Asp_protease_2,RVT_1,RT_RNaseH,Integrase_H2C2

	BLFJ01003147.1:65191_69652	4461	RT_RNaseH_2
	BLFJ01003254.1:132183_137597	5414	Asp_protease_2,RVT_1,RT_RNaseH_2,Integrase_H2C2
	BLFJ01003435.1:640260_645824	5564	RT_RNaseH_2
	BLFJ01004422.1:75085_80583	5498	gag-asp_proteas,RT_RNaseH_2,Integrase_H2C2
	BLFJ01004471.1:124434_129160	4726	Asp_protease_2,RT_RNaseH_2,rve_3
	BLFJ01005715.1:289776_294932	5156	Asp_protease_2,RT_RNaseH_2
	BLFJ01006147.1:647422_651865	4443	gag-asp_proteas,RT_RNaseH
	BLFJ01006410.1:310358_320524	10166	RT_RNaseH_2,SUa-2TM
	BLFJ01006734.1:17227_18664	1437	RVT_1
Acropora nasuta	BLFL01000190.1:987058_992651	5593	Asp_protease_2,RT_RNaseH,Integrase_H2C2
	BLFL01000586.1:991670_997252	5582	RVT_1,RT_RNaseH_2,Integrase_H2C2
	BLFL01001418.1:1215370_1219150	3780	Asp_protease_2,RVT_1,RT_RNaseH_2
	BLFL01001912.1:128185_133632	5447	Asp_protease_2,RVT_1,RT_RNaseH_2,rve_3
	BLFL01002071.1:924145_930897	6752	Asp_protease_2,RVT_1,RT_RNaseH_2
	BLFL01003899.1:17569_23181	5612	gag-asp_proteas,RVT_1,RT_RNaseH_2,Integrase_H2C2
	BLFL01004182.1:119692_125231	5539	Asp_protease_2,RT_RNaseH_2,Integrase_H2C2
Acropora selago	BLFM01000339.1:77536_83107	5571	Asp_protease_2,RVT_1,RT_RNaseH_2,Integrase_H2C2
	BLFM01000737.1:12256_17993	5737	gag-asp_proteas,RT_RNaseH_2
	BLFM01000793.1:286626_292199	5573	gag-asp_proteas,RVT_1,RT_RNaseH_2,Integrase_H2C2
	BLFM01001625.1:577386_582810	5424	Asp_protease_2,RVT_1,RT_RNaseH,Integrase_H2C2,rve_3
	BLFM01001694.1:236658_242498	5840	Asp_protease_2,RT_RNaseH_2,Integrase_H2C2
	BLFM01001826.1:1212933_1218492	5559	gag-asp_proteas,RT_RNaseH_2,Integrase_H2C2
	BLFM01002510.1:32655_38154	5499	Asp_protease_2,RVT_1,RVT_1,RT_RNaseH_2
	BLFM01002627.1:229758_234879	5121	Asp_protease_2,RVT_1
	BLFM01003472.1:207905_213520	5615	Asp_protease_2,RVT_1,RT_RNaseH_2,Integrase_H2C2
	BLFM01004689.1:143635_151449	7814	gag-asp_proteas,RVT_1,RT_RNaseH_2,Integrase_H2C2
	BLFM01005261.1:117981_123542	5561	gag-asp_proteas,RVT_1,RT_RNaseH_2,Integrase_H2C2
	BLFM01005681.1:35261_40640	5379	Asp_protease_2,RVT_1,RT_RNaseH,Integrase_H2C2
	BLFM01005707.1:524302_531664	7362	RVT_1,RT_RNaseH_2,zf-AD

Acropora tenuis	BLAZ01000046.1:283779_289421	5642	gag-asp_proteas,RT_RNaseH
	BLAZ01000146.1:12641_17492	4851	RVT_1,RT_RNaseH_2
	BLAZ01000473.1:974072_979589	5517	Asp_protease_2,RVT_1,RT_RNaseH_2
	BLAZ01000473.1:2505085_2510722	5637	Asp_protease_2,RVT_1,RT_RNaseH_2,Integrase_H2C2
	BLAZ01000724.1:282372_287929	5557	Asp_protease_2,RVT_1,RT_RNaseH_2,rve_3
	BLAZ01000782.1:273481_278867	5386	Asp_protease_2,RVT_1,RT_RNaseH_2,RT_RNaseH,rve_3
	BLAZ01000797.1:470015_475541	5526	Asp_protease_2,RT_RNaseH_2,Integrase_H2C2
	BLAZ01000808.1:697012_702015	5003	RVT_1,RT_RNaseH_2,rve_3
	BLAZ01000876.1:32824_38461	5637	gag-asp_proteas,RVT_1,RT_RNaseH_2,Integrase_H2C2
	BLAZ01000913.1:48432_54103	5671	Asp_protease_2,RVT_1,RT_RNaseH_2,Integrase_H2C2
	BLAZ01001035.1:701647_707156	5509	zf-RVT,Asp_protease_2,RVT_1,RT_RNaseH_2,Integrase_H2C2,rve_3
	BLAZ01001171.1:343041_356967	13926	gag-asp_proteas,RT_RNaseH,DDE_3
Acropora yongei	BLFN01000113.1:450601_456159	5558	Asp_protease_2,RVT_1,RT_RNaseH_2,Integrase_H2C2
	BLFN01000113.1:561604_572343	10739	Phasin,gag-asp_proteas,RT_RNaseH,Integrase_H2C2
	BLFN01000113.1:4983784_4990396	6612	Asp_protease_2,RVT_1,RT_RNaseH,Integrase_H2C2
	BLFN01000122.1:354751_360465	5714	gag-asp_proteas,RVT_1,RT_RNaseH,Integrase_H2C2
	BLFN01000134.1:1277266_1282828	5562	Asp_protease_2,RVT_1,RT_RNaseH_2,Integrase_H2C2
	BLFN01000134.1:3046949_3062002	15053	Spc24,Exo_endo_phos_2,RVT_1,RVT_1
	BLFN01000134.1:3815578_3821014	5436	gag-asp_proteas,RVT_1,RT_RNaseH_2,Integrase_H2C2
	BLFN01000162.1:296240_301570	5330	RT_RNaseH_2,Integrase_H2C2
	BLFN01000190.1:163576_169185	5609	gag-asp_proteas,RVT_1,RT_RNaseH_2,Integrase_H2C2
	BLFN01000196.1:3963915_3969520	5605	gag-asp_proteas,RVT_1,RT_RNaseH_2,Integrase_H2C2
	BLFN01000199.1:298312_303952	5640	gag-asp_proteas,RVT_1,RT_RNaseH_2,Integrase_H2C2
	BLFN01000199.1:4766569_4772130	5561	Asp_protease_2,RVT_1,RT_RNaseH_2,Integrase_H2C2
	BLFN01000201.1:578664_584297	5633	RVT_1,RT_RNaseH_2,Integrase_H2C2
	BLFN01000201.1:2863650_2869307	5657	Asp_protease_2,RT_RNaseH_2,Integrase_H2C2
	BLFN01000227.1:1501727_1507277	5550	RT_RNaseH_2,Integrase_H2C2
	BLFN01000266.1:1231332_1236336	5004	RVT_1,RT_RNaseH_2,rve_3

	BLFN01000290.1:7408276_7417382	9106	Asp_protease_2,RVT_1,RT_RNaseH_2,Integrase_H2C2,rve
	BLFN01000326.1:920643_926165	5522	gag-asp_proteas,RVT_1,RT_RNaseH_2,Integrase_H2C2
	BLFN01000410.1:81916_87610	5694	Asp_protease_2,RVT_1,RT_RNaseH_2,Integrase_H2C2
	BLFN01000419.1:434915_443876	8961	Asp_protease_2,RVT_1,RT_RNaseH_2,RT_RNaseH,Integrase_H2C2,rve_3,Exo_endo_phos_2,MREG,RVT_1,RVT_1
	BLFN01000429.1:980060_988340	8280	RVT_1,RT_RNaseH_2,Integrase_H2C2
	BLFN01000432.1:198160_203789	5629	gag-asp_proteas,RVT_1,RT_RNaseH_2,Integrase_H2C2
	BLFN01000547.1:396755_402300	5545	gag-asp_proteas,RVT_1,RT_RNaseH_2,Integrase_H2C2
	BLFN01000574.1:1453871_1459431	5560	Asp_protease_2,RVT_1,RT_RNaseH,Integrase_H2C2
	BLFN01000578.1:25108_30779	5671	Asp_protease_2,RVT_1,RT_RNaseH_2
	BLFN01000617.1:500848_506352	5504	RVT_1,RT_RNaseH_2,Integrase_H2C2,rve_3
	BLFN01000617.1:2960404_2966015	5611	gag-asp_proteas,RVT_1,RT_RNaseH,Integrase_H2C2
	BLFN01000617.1:7707311_7712815	5504	gag-asp_proteas,RVT_1,RT_RNaseH_2,Integrase_H2C2
	BLFN01000640.1:395502_401132	5630	gag-asp_proteas,RVT_1,RT_RNaseH_2,Integrase_H2C2
	BLFN01000740.1:1811820_1817505	5685	Asp_protease_2,RVT_1,RT_RNaseH_2,Integrase_H2C2
	BLFN01000866.1:1525166_1530692	5526	Asp_protease_2,RT_RNaseH_2,Integrase_H2C2
	BLFN01000880.1:1075142_1087405	12263	Asp_protease_2,RVT_1,RT_RNaseH_2
	BLFN01000897.1:530807_535961	5154	Asp_protease_2,RVT_1,RT_RNaseH_2,Integrase_H2C2
	BLFN01000917.1:3372073_3376367	4294	Asp_protease_2,RVT_1,RT_RNaseH_2
	BLFN01000960.1:416923_422606	5683	Asp_protease_2,RVT_1,RT_RNaseH_2,Integrase_H2C2
	WHPX01000129.1:49538_55100	5562	MADF_DNA_bdg
	WHPX01000164.1:71836_77406	5570	Asp_protease_2,RVT_1,RT_RNaseH_2,Integrase_H2C2
	WHPX01000263.1:260177_269084	8907	gag-asp_proteas,RVT_1,RT_RNaseH_2,Integrase_H2C2
	WHPX01000276.1:10379_15879	5500	gag-asp_proteas,RVT_1,RT_RNaseH_2,Integrase_H2C2
Actinia equina	WHPX01000316.1:490982_496548	5566	gag-asp_proteas,RVT_1,RT_RNaseH_2,Integrase_H2C2
	WHPX01000366.1:133898_139450	5552	Asp_protease_2,RVT_1,RT_RNaseH_2,Integrase_H2C2
	WHPX01000369.1:40707_46283	5576	gag-asp_proteas,RVT_1,RT_RNaseH_2,Integrase_H2C2
	WHPX01000405.1:104846_11040	5562	gag-asp_proteas,RVT_1,RT_RNaseH_2,Integrase_H2C2

	8		
	WHPX01000446.1:87240_92807	5567	gag-asp_proteas,RVT_1,RT_RNaseH_2,Integrase_H2C2
	WHPX01000583.1:415238_420792	5554	gag-asp_proteas,RVT_1,RT_RNaseH_2,Integrase_H2C2
	WHPX01000797.1:101688_107242	5554	gag-asp_proteas,RVT_1,RT_RNaseH_2,Integrase_H2C2
	WHPX01000835.1:82231_87801	5570	gag-asp_proteas,RVT_1,RT_RNaseH_2,Integrase_H2C2
	WHPX01000897.1:236387_241936	5549	gag-asp_proteas,RVT_1,RT_RNaseH_2,Integrase_H2C2
	WHPX01000969.1:567243_572797	5554	gag-asp_proteas,RVT_1,RT_RNaseH_2,Integrase_H2C2
	WHPX01001049.1:243849_249417	5568	gag-asp_proteas,RVT_1,RT_RNaseH_2,Integrase_H2C2
	WHPX01001086.1:241174_246728	5554	gag-asp_proteas,RVT_1,RT_RNaseH_2,Integrase_H2C2
	WHPX01001086.1:357132_363047	5915	HJURP_C
	WHPX01001123.1:806989_812561	5572	gag-asp_proteas,RVT_1,RT_RNaseH_2,Integrase_H2C2
	WHPX01001125.1:328892_335382	6490	gag-asp_proteas,RVT_1,RT_RNaseH_2,Integrase_H2C2
	WHPX01001206.1:7113_12686	5573	gag-asp_proteas,RVT_1,RT_RNaseH_2,Integrase_H2C2
	WHPX01001294.1:44001_49566	5565	gag-asp_proteas,RVT_1,RT_RNaseH_2,Integrase_H2C2
	WHPX01001306.1:182707_188278	5571	gag-asp_proteas,RVT_1,RT_RNaseH_2,Integrase_H2C2
	WHPX01001328.1:387365_392924	5559	gag-asp_proteas,RVT_1,RT_RNaseH_2,Integrase_H2C2
Astreopora myriophthalma	BLFK01000034.1:1151547_1157169	5622	gag-asp_proteas,RVT_1,RT_RNaseH_2,Integrase_H2C2
	BLFK01000113.1:3908457_3913994	5537	Asp_protease_2,RVT_1,RT_RNaseH_2,Integrase_H2C2
	BLFK01000444.1:2299282_2304829	5547	Asp_protease_2,RVT_1,RT_RNaseH_2,Integrase_H2C2
	BLFK01000448.1:880559_886039	5480	gag-asp_proteas,RVT_1,RT_RNaseH_2,Integrase_H2C2
	BLFK01000457.1:48506_54059	5553	Asp_protease_2,RVT_1,RT_RNaseH_2,Integrase_H2C2
	BLFK01000664.1:116604_123109	6505	RVT_1,RT_RNaseH_2,Integrase_H2C2
	BLFK01000695.1:449177_454655	5478	gag-asp_proteas,RT_RNaseH,Integrase_H2C2
	BLFK01000699.1:77240_83285	6045	gag-asp_proteas,RVT_1,RT_RNaseH_2,Integrase_H2C2
	BLFK01000748.1:1817820_18239	6151	gag-asp_proteas,RVT_1,Integrase_H2C2

	71		
	BLFK01000815.1:742277_747820	5543	Asp_protease_2,RVT_1,RT_RNaseH_2,Integrase_H2C2
	BLFK01000874.1:218685_224161	5476	gag-asp_proteas,RVT_1,RT_RNaseH_2,Integrase_H2C2
	BLFK01000947.1:71215_76346	5131	gag-asp_proteas,RVT_1,RT_RNaseH
	BLFK01001047.1:761237_766829	5592	Asp_protease_2,RVT_1,RT_RNaseH_2,Integrase_H2C2
	BLFK01001048.1:683242_688890	5648	gag-asp_proteas,RVT_1,RT_RNaseH_2,Integrase_H2C2
	BLFK01001048.1:1105050_1111827	6777	Asp_protease_2,RVT_1,RT_RNaseH,Integrase_H2C2
	BLFK01001060.1:310155_315779	5624	Asp_protease_2,RT_RNaseH_2,Integrase_H2C2
	BLFK01001060.1:385034_391047	6013	gag-asp_proteas,RVT_1,RT_RNaseH_2,Integrase_H2C2
	BLFK01001065.1:38288_43865	5577	gag-asp_proteas,RVT_1,RT_RNaseH_2,Integrase_H2C2
Cassiopea xamachana	OLMO01004854.1:23079_29884	6805	gag-asp_proteas,UPF0220,RVT_1,RT_RNaseH_2,Integrase_H2C2
Clytia hemisphaerica	CACVBU010000079.1:136480_142010	5530	RVT_1,RT_RNaseH_2,Integrase_H2C2
	CACVBU010000089.1:61185_66627	5442	gag-asp_proteas,RVT_1,RT_RNaseH_2
	CACVBU010000116.1:58898_64395	5497	gag-asp_proteas,RVT_1,RT_RNaseH_2,Integrase_H2C2,rve
	CACVBU010000237.1:703777_709206	5429	gag-asp_proteas,RVT_1,RT_RNaseH_2,Integrase_H2C2,rve
	CACVBU010000250.1:609068_614349	5281	Asp_protease_2,RVT_1,RT_RNaseH_2
	CACVBU010000255.1:72960_78401	5441	gag-asp_proteas,RVT_1,RT_RNaseH_2,FrhB_FdhB_C
	CACVBU010000261.1:210884_216148	5264	gag-asp_proteas,RVT_1,RT_RNaseH_2
	CACVBU010000468.1:259067_264424	5357	gag-asp_proteas,RVT_1,RT_RNaseH_2,Integrase_H2C2,rve
	CACVBU010000501.1:491123_496618	5495	gag-asp_proteas,RVT_1,RT_RNaseH_2,Integrase_H2C2,rve
	CACVBU010000525.1:881501_887066	5565	gag-asp_proteas,RVT_1,RT_RNaseH_2,Integrase_H2C2
	CACVBU010000598.1:63890_69454	5564	RVT_1,RT_RNaseH_2,Integrase_H2C2
	CACVBU010000599.1:26252_31917	5665	gag-asp_proteas,RVT_1,RT_RNaseH_2,Integrase_H2C2,Integrase_H2C2,VasL
	CACVBU010000656.1:586625_592034	5409	gag-asp_proteas,RVT_1,RT_RNaseH_2,FrhB_FdhB_C

	CACVBU010000784.1:581725_587168	5443	gag-asp_proteas,RVT_1,RT_RNaseH_2,Integrase_H2C2,rve
	CACVBU010000791.1:397823_405423	7600	gag-asp_proteas,RVT_1
	CACVBU010000807.1:177764_183200	5436	gag-asp_proteas,RVT_1,RT_RNaseH_2
	CACVBU010000821.1:538523_543591	5068	gag-asp_proteas,RVT_1,RT_RNaseH_2,Integrase_H2C2
	CACVBU010000849.1:60238_65671	5433	gag-asp_proteas,RVT_1,RT_RNaseH_2,FrhB_FdhB_C,DUF3155
	CACVBU010000902.1:433991_438132	4141	RT_RNaseH_2,Integrase_H2C2
	CACVBU010000971.1:1383016_1395936	12920	gag-asp_proteas,RVT_1,RT_RNaseH_2,Integrase_H2C2,rve
	CACVBU010001002.1:871955_877296	5341	gag-asp_proteas,RVT_1,RT_RNaseH_2,Integrase_H2C2,rve
	CACVBU010001089.1:38280_43607	5327	gag-asp_proteas,RVT_1,RT_RNaseH_2,Integrase_H2C2,rve
	CACVBU010001174.1:109471_114187	4716	gag-asp_proteas,RVT_1,RT_RNaseH_2,Integrase_H2C2
	CACVBU010001190.1:286209_289608	3399	RVT_1,RT_RNaseH_2,VasL
	CACVBU010001240.1:365182_370538	5356	gag-asp_proteas,RVT_1,RT_RNaseH_2,Integrase_H2C2,rve
	CACVBU010001258.1:360207_365523	5316	gag-asp_proteas,RVT_1,RT_RNaseH_2,Integrase_H2C2
	CACVBU010001292.1:667169_671718	4549	RVT_1,RT_RNaseH_2,Integrase_H2C2
	CACVBU010001311.1:320382_325801	5419	gag-asp_proteas,RVT_1,RT_RNaseH_2
	CACVBU010001320.1:113344_118721	5377	RVT_1,RT_RNaseH_2,Integrase_H2C2
	CACVBU010001323.1:13277_19715	6438	RVT_1,RT_RNaseH_2,Integrase_H2C2
	CACVBU010001363.1:317224_322736	5512	gag-asp_proteas,RVT_1,RT_RNaseH_2,Integrase_H2C2,rve
Heteractis crispa	JAABNW010000723.1:16214745_16219975	5230	RVT_1,RT_RNaseH,Integrase_H2C2,rve
Montipora	BLFO01000007.1:831128_838306	7178	RT_RNaseH,Integrase_H2C2

cactus	BLFO01000031.1:30127_38104	7977	gag-asp_proteas,RVT_1,Integrase_H2C2
	BLFO01000082.1:15802_23684	7882	gag-asp_proteas,RVT_1,Integrase_H2C2,zf-H2C2
	BLFO01000215.1:918088_924215	6127	Asp_protease_2,RVT_1,RT_RNaseH_2,Integrase_H2C2
	BLFO01000215.1:1251706_1258974	7268	gag-asp_proteas,RVT_1,RVT_1,RT_RNaseH_2,Integrase_H2C2
	BLFO01000850.1:122740_128393	5653	gag-asp_proteas,RVT_1,RT_RNaseH_2,Integrase_H2C2
	BLFO01000876.1:371591_377082	5491	Asp_protease_2,RVT_1,RT_RNaseH_2,RT_RNaseH,Integrase_H2C2
	BLFO01001436.1:8928_14841	5913	Asp_protease_2,RVT_1,RT_RNaseH_2,Integrase_H2C2
	BLFO01001520.1:1378538_1386544	8006	RVT_1,RT_RNaseH_2,Integrase_H2C2
	BLFO01001746.1:265089_270686	5597	gag-asp_proteas,RVT_1,RT_RNaseH_2,Integrase_H2C2
	BLFO01001776.1:146185_149740	3555	RT_RNaseH_2,Integrase_H2C2
	BLFO01001896.1:258920_266192	7272	Exo_endo_phos_2,Asp_protease_2,RVT_1,RT_RNaseH_2,Exo_endo_phos_2
	BLFO01001913.1:18728_26210	7482	Asp_protease_2,RVT_1,RT_RNaseH_2,Integrase_H2C2
	BLFO01002439.1:211140_219712	8572	gag-asp_proteas,RT_RNaseH_2,Integrase_H2C2
	BLFO01002439.1:605141_619861	14720	gag-asp_proteas,RVT_1,RT_RNaseH_2
	BLFO01002590.1:56328_63677	7349	RT_RNaseH_2,Integrase_H2C2
	BLFO01002768.1:310638_316706	6068	RT_RNaseH_2,Integrase_H2C2,rve
	BLFO01002873.1:18759_24386	5627	Asp_protease_2,RT_RNaseH_2,Integrase_H2C2
	BLFO01002896.1:581663_587200	5537	Asp_protease_2,RVT_1,RT_RNaseH_2,Integrase_H2C2,rve
	BLFO01003116.1:478193_483129	4936	RT_RNaseH,Integrase_H2C2
	BLFO01003213.1:28222_33795	5573	Asp_protease_2,RVT_1,RT_RNaseH_2
	BLFO01003511.1:493224_501388	8164	gag-asp_proteas,RVT_1,RT_RNaseH_2,Integrase_H2C2,rve,Exo_endo_phos_2,RVT_1
	BLFO01004070.1:1269659_1273955	4296	RT_RNaseH_2,Integrase_H2C2
	BLFO01004425.1:135655_140452	4797	Asp_protease_2,RVT_1,RT_RNaseH,Integrase_H2C2
	BLFO01004439.1:494691_500285	5594	Asp_protease_2,RVT_1,RT_RNaseH_2,Integrase_H2C2
	BLFO01004489.1:746045_752687	6642	RT_RNaseH,Integrase_H2C2
	BLFO01004705.1:307246_312827	5581	Asp_protease_2,RVT_1,RT_RNaseH_2,Integrase_H2C2
	BLFO01004860.1:389445_394952	5507	gag-asp_proteas,RVT_1,RT_RNaseH_2,rve
Montipora capitata	RDEB01000061.1:590270_595671	5401	gag-asp_proteas,RVT_1,RT_RNaseH_2,Integrase_H2C2
	RDEB01000125.1:315818_319821	4003	RVT_1,RT_RNaseH,Integrase_H2C2
	RDEB01000133.1:357947_36363	5687	Asp_protease_2,RVT_1,RT_RNaseH_2,Integrase_H2C2

	4		
	RDEB01000157.1:341187_346365	5178	gag-asp_proteas,RVT_1,RT_RNaseH_2,Integrase_H2C2
	RDEB01000208.1:214533_219911	5378	RVT_1,RVT_1,RT_RNaseH_2
	RDEB01000258.1:7304_12154	4850	Asp_protease_2,RVT_1,Integrase_H2C2,rve
	RDEB01000429.1:146172_151659	5487	RT_RNaseH_2,Integrase_H2C2,zf-H2C2
	RDEB01000470.1:186729_192255	5526	Asp_protease_2,RVT_1,RT_RNaseH_2,Integrase_H2C2,rve
	RDEB01000597.1:186868_192669	5801	Asp_protease_2,RVT_1,RT_RNaseH_2,Integrase_H2C2,rve
	RDEB01002456.1:9389_13763	4374	Asp_protease_2,RVT_1,RT_RNaseH_2,Integrase_H2C2
	RDEB01002571.1:6219_11738	5519	TetR_C_24,Asp_protease_2,TetR_C_24,RVT_1,RVT_1,RT_RNaseH_2,Integrase_H2C2,rve
	RDEB01003853.1:3796_9420	5624	gag-asp_proteas,RVT_1,RT_RNaseH_2,Integrase_H2C2
	RDEB01009054.1:103990_109458	5468	Asp_protease_2,RVT_1,RT_RNaseH_2,Integrase_H2C2
	RDEB01009840.1:6640_12120	5480	RVT_1,RT_RNaseH,Integrase_H2C2
	RDEB01025049.1:15754_21191	5437	RVT_1,RT_RNaseH,Integrase_H2C2
Montipora efflorescens	BLFP01000100.1:3252742_3256344	3602	Asp_protease_2
	BLFP01000213.1:34868_43112	8244	gag-asp_proteas,RVT_1,Integrase_H2C2
	BLFP01000651.1:91403_97083	5680	Asp_protease_2,RVT_1,RT_RNaseH_2,Integrase_H2C2
	BLFP01000791.1:465391_469614	4223	RT_RNaseH_2,Integrase_H2C2
	BLFP01000793.1:639549_645061	5512	TetR_C_24,Asp_protease_2,TetR_C_24,RVT_1,RT_RNaseH_2
	BLFP01000892.1:1481639_1488101	6462	gag-asp_proteas,RVT_1,RT_RNaseH_2
	BLFP01001209.1:26592_32209	5617	Asp_protease_2,RVT_1,RT_RNaseH_2
	BLFP01001286.1:2471112_2476357	5245	RVT_1,Integrase_H2C2
	BLFP01001708.1:3829847_3843645	13798	RVT_1,RVT_1
	BLFP01001839.1:144289_151629	7340	Asp_protease_2,RVT_1,RT_RNaseH_2,Integrase_H2C2
	BLFP01001839.1:1353723_1359212	5489	Asp_protease_2,RVT_1,RT_RNaseH_2,Integrase_H2C2
	BLFP01002247.1:1822785_1836820	14035	gag-asp_proteas,RVT_1,RT_RNaseH_2
	BLFP01002553.1:733272_738568	5296	gag-asp_proteas,RVT_1,Integrase_H2C2
	BLFP01002823.1:1734937_17497	14810	ResIII,ResIII,ResIII,gag-asp_proteas,RVT_1,RT_RNaseH_

	47		2,Integrase_H2C2
	BLFP01003613.1:384459_388376	3917	RT_RNaseH_2,Integrase_H2C2
	BLFP01003634.1:99125_105837	6712	gag-asp_proteas,RVT_1,RT_RNaseH_2
	BLFP01003638.1:926143_934150	8007	RVT_1,gag-asp_proteas,RT_RNaseH_2,Integrase_H2C2,VKG_Carbox
	BLFP01003780.1:117598_126216	8618	RVT_1,RT_RNaseH,DUF347,RVT_1
	BLFP01004184.1:1666063_1671384	5321	gag-asp_proteas,RVT_1,Integrase_H2C2,zf-H2C2
	BLFP01004354.1:687779_693468	5689	Asp_protease_2,RVT_1,RT_RNaseH_2,Integrase_H2C2
	BLFP01004641.1:107998_113600	5602	gag-asp_proteas,RVT_1,RT_RNaseH_2,Integrase_H2C2
	BLFP01004765.1:387319_389129	1810	
	BLFP01004924.1:314874_319624	4750	RVT_1,RT_RNaseH,Integrase_H2C2
Morbakka virulenta	RDPX01000001.1:4030212_4035607	5395	MADF_DNA_bdg,gag-asp_proteas,RVT_1,RVT_1,RT_RNaseH_2,Integrase_H2C2,RVT_1
	RDPX01000115.1:1995158_1996473	1315	
	RDPX01000115.1:2249823_2255155	5332	gag-asp_proteas,RVT_1,RT_RNaseH_2,Integrase_H2C2,rve
	RDPX01001166.1:6916_11530	4614	RT_RNaseH_2,RT_RNaseH
	RDPX01000120.1:1080758_1087722	6964	RT_RNaseH_2,PHD
	RDPX01000122.1:1477549_1482914	5365	gag-asp_proteas,RVT_1,RT_RNaseH_2,Integrase_H2C2,rve
	RDPX01000123.1:87758_93092	5334	gag-asp_proteas,RVT_1,RT_RNaseH_2,Integrase_H2C2,rve
	RDPX01000129.1:2310069_2315386	5317	gag-asp_proteas,RVT_1,RT_RNaseH_2,Integrase_H2C2,rve
	RDPX01000131.1:497698_503822	6124	gag-asp_proteas,RVT_1,RVT_1,RT_RNaseH_2
	RDPX01000132.1:320267_325570	5303	zf-RVT,gag-asp_proteas,RVT_1,RT_RNaseH_2,Integrase_H2C2
	RDPX01000015.1:5393909_5398499	4590	gag-asp_proteas,RVT_1,RT_RNaseH_2
	RDPX01000156.1:789844_794853	5009	gag-asp_proteas,RVT_1,RT_RNaseH
	RDPX01000017.1:3924867_3928986	4119	gag-asp_proteas,RVT_1,RT_RNaseH_2
	RDPX01000199.1:332693_335662	2969	DUF5584

	RDPX01000209.1:474849_484216	9367	RVT_1,RT_RNaseH,Integrase_H2C2,THAP,HTH_Tnp_4,DDE_Tnp_4
	RDPX01000227.1:277635_282908	5273	gag-asp_proteas,RVT_1,RT_RNaseH_2,Integrase_H2C2,rve
	RDPX01000232.1:846808_851863	5055	gag-asp_proteas,RT_RNaseH_2,RT_RNaseH,Integrase_H2C2
	RDPX01000024.1:2507766_2513035	5269	gag-asp_proteas,RVT_1,RT_RNaseH_2,Integrase_H2C2
	RDPX01000026.1:142617_147927	5310	gag-asp_proteas,RVT_1,RT_RNaseH_2,Integrase_H2C2,rve
	RDPX01000270.1:69225_74613	5388	Asp_protease_2,RVT_1,RT_RNaseH_2,RT_RNaseH,Integrase_H2C2
	RDPX01000028.1:1261885_1267200	5315	gag-asp_proteas,RVT_1,RT_RNaseH_2,Integrase_H2C2,rve
	RDPX01000287.1:549711_555292	5581	DUF5690
	RDPX01000288.1:64109_69469	5360	gag-asp_proteas,RVT_1,RT_RNaseH,Integrase_H2C2
	RDPX01000289.1:575814_587433	11619	
	RDPX01000294.1:603832_609620	5788	gag-asp_proteas,RT_RNaseH,Integrase_H2C2
	RDPX01000299.1:826173_830582	4409	gag-asp_proteas,Integrase_H2C2,rve
	RDPX01000362.1:433809_439166	5357	gag-asp_proteas,RVT_1,RT_RNaseH_2,Integrase_H2C2,rve
	RDPX01000403.1:308827_314158	5331	gag-asp_proteas,RVT_1,RT_RNaseH_2,Integrase_H2C2,rve
	RDPX01000041.1:1970620_1981179	10559	RVT_1,RT_RNaseH_2
	RDPX01000042.1:591496_596760	5264	gag-asp_proteas,RVT_1,RT_RNaseH_2
	RDPX01000042.1:2327158_2335369	8211	zf-RVT,gag-asp_proteas,Exo_endo_phos,RVT_1
	RDPX01000446.1:438567_443969	5402	Asp_protease_2,RVT_1,RT_RNaseH_2
	RDPX01000047.1:2779875_2785302	5427	RVT_1,Integrase_H2C2
	RDPX01000005.1:2602464_2606276	3812	zf-RVT,gag-asp_proteas,dUTPase,dUTPase
	RDPX01000050.1:773035_77846	5432	RVT_1,RT_RNaseH_2,Integrase_H2C2

	7		
	RDPX01000507.1:189042_194388	5346	gag-asp_proteas,RVT_1,RT_RNaseH_2,Integrase_H2C2,rve
	RDPX01000558.1:166515_171951	5436	RVT_1,RT_RNaseH_2,Integrase_H2C2
	RDPX01000059.1:1975589_1989915	14326	
	RDPX01000593.1:109959_115531	5572	gag-asp_proteas,RVT_1,RT_RNaseH_2,Integrase_H2C2,rve
	RDPX01000608.1:2109_9902	7793	RVT_1,RT_RNaseH,RVT_1
	RDPX01000078.1:1914275_1923710	9435	RVT_1,RVT_1,RVT_1
	RDPX01000081.1:734560_739876	5316	gag-asp_proteas,RVT_1,RT_RNaseH_2,Integrase_H2C2,rve
	RDPX01000089.1:4304_8330	4026	gag-asp_proteas,RVT_1,RT_RNaseH_2,Astro_capsid_p
	RDPX01000090.1:2367342_2372703	5361	gag-asp_proteas,RVT_1,RT_RNaseH_2,Integrase_H2C2,rve
	RDPX01000092.1:486608_491932	5324	gag-asp_proteas,RVT_1,RT_RNaseH_2,Integrase_H2C2,rve
	RDPX01000098.1:2288974_2292340	3366	
Nemopilema nomurai	ML133411.1:256935_262288	5353	RVT_1,RT_RNaseH_2,Integrase_H2C2
	ML133502.1:1978237_1983590	5353	gag-asp_proteas,RVT_1,RT_RNaseH_2,Integrase_H2C2
Pachyseris speciosa	JAEMWC010000002.1:283260_288792	5532	Asp_protease_2,RVT_1,RT_RNaseH_2,Integrase_H2C2,rve
	JAEMWC010000003.1:1382627_1388308	5681	RVT_1,RT_RNaseH_2,Integrase_H2C2
	JAEMWC010000005.1:945351_950873	5522	Asp_protease_2,RVT_1,RT_RNaseH_2,Integrase_H2C2,rve
	JAEMWC010000005.1:2298772_2305827	7055	RVT_1,RT_RNaseH_2,Integrase_H2C2,rve
	JAEMWC010000010.1:2410025_2415630	5605	gag-asp_proteas,RVT_1,RT_RNaseH_2,Integrase_H2C2
	JAEMWC010000015.1:2452453_2458133	5680	gag-asp_proteas,RVT_1,RT_RNaseH_2,Integrase_H2C2
	JAEMWC010000017.1:305992_311594	5602	Asp_protease_2,RVT_1,RT_RNaseH_2
	JAEMWC010000024.1:1916126_1921615	5489	RVT_1,RT_RNaseH_2,Integrase_H2C2

	JAEMWC010000027.1:1401526_1407074	5548	gag-asp_proteas,RVT_1,RT_RNaseH_2,Integrase_H2C2,rve
	JAEMWC010000032.1:1340841_1346532	5691	Asp_protease_2,RVT_1,RT_RNaseH_2,Integrase_H2C2
	JAEMWC010000033.1:7205_12884	5679	Asp_protease_2,RVT_1,RT_RNaseH_2,Integrase_H2C2
	JAEMWC010000035.1:937283_942808	5525	TetR_C_24,Asp_protease_2,TetR_C_24,RVT_1,RT_RNaseH_2,rve
	JAEMWC010000037.1:158753_164151	5398	gag-asp_proteas,RT_RNaseH_2
	JAEMWC010000046.1:1908035_1913575	5540	TetR_C_24,Asp_protease_2,TetR_C_24,RVT_1,RT_RNaseH_2,Integrase_H2C2,rve
	JAEMWC010000047.1:363592_369149	5557	RVT_1,RT_RNaseH_2,zf-H2C2
	JAEMWC010000050.1:1436144_1441680	5536	TetR_C_24,Asp_protease_2,TetR_C_24,RVT_1,RT_RNaseH_2,Integrase_H2C2,rve
	JAEMWC010000060.1:250058_259277	9219	gag-asp_proteas,RVT_1,RT_RNaseH_2,Integrase_H2C2
	JAEMWC010000065.1:1504906_1510672	5766	Asp_protease_2,RT_RNaseH_2,Integrase_H2C2
	JAEMWC010000066.1:78624_84131	5507	gag-asp_proteas,RVT_1,RT_RNaseH_2,Integrase_H2C2
	JAEMWC010000069.1:161439_167153	5714	Asp_protease_2,RVT_1,RT_RNaseH_2,Integrase_H2C2
	JAEMWC010000078.1:242115_247713	5598	RT_RNaseH_2,RT_RNaseH,zf-H2C2
	JAEMWC010000081.1:1466871_1472577	5706	Asp_protease_2,RVT_1,RT_RNaseH_2,Integrase_H2C2
	JAEMWC010000085.1:1103036_1109754	6718	gag-asp_proteas,RVT_1,RT_RNaseH_2,Integrase_H2C2,zf-RVT
	JAEMWC010000088.1:1561830_1568941	7111	RVT_1,RVT_1,DDE_Tnp_1_7,DDE_Tnp_1_7,RT_RNaseH_2,Integrase_H2C2
	JAEMWC010000092.1:1387627_1393134	5507	RT_RNaseH_2,Integrase_H2C2
	JAEMWC010000106.1:1029486_1035039	5553	TetR_C_24,Asp_protease_2,TetR_C_24,RVT_1,RT_RNaseH_2,Integrase_H2C2,rve
	JAEMWC010000112.1:781272_786794	5522	gag-asp_proteas,RVT_1,RT_RNaseH_2,Integrase_H2C2
	JAEMWC010000113.1:387392_3	5638	gag-asp_proteas,RVT_1,RT_RNaseH_2

	93030		
	JAEMWC010000115.1:1376277_1385623	9346	RVT_1,Integrase_H2C2,zf-H2C2
	JAEMWC010000116.1:151250_156902	5652	gag-asp_proteas,RVT_1,RT_RNaseH_2,Integrase_H2C2
	JAEMWC010000116.1:601214_606845	5631	Asp_protease_2,RVT_1,RT_RNaseH_2,Integrase_H2C2
	JAEMWC010000124.1:1140768_1146284	5516	TetR_C_24,Asp_protease_2,TetR_C_24,RVT_1,RT_RNaseH_2,Integrase_H2C2,rve
	JAEMWC010000127.1:533964_546732	12768	Asp_protease_2,RVT_1,RT_RNaseH_2,zf-H2C2
	JAEMWC010000133.1:699542_710090	10548	Asp_protease_2,RVT_1,RT_RNaseH_2,Integrase_H2C2
	JAEMWC010000138.1:474294_479949	5655	gag-asp_proteas,RVT_1,RT_RNaseH_2,Integrase_H2C2
	JAEMWC010000138.1:861901_873738	11837	DDE_Tnp_IS1595,Asp_protease_2,RVT_1,RT_RNaseH_2,Integrase_H2C2
	JAEMWC010000141.1:460432_466017	5585	Asp_protease_2,RVT_1,RT_RNaseH_2,zf-H2C2
	JAEMWC010000159.1:209182_214785	5603	Asp_protease_2,RVT_1,RT_RNaseH_2,zf-H2C2
	JAEMWC010000170.1:132440_142007	9567	gag-asp_proteas,RVT_1,RT_RNaseH_2,Integrase_H2C2
	JAEMWC010000170.1:1155835_1161258	5423	Asp_protease_2,RT_RNaseH,zf-H2C2
	JAEMWC010000176.1:763586_769200	5614	Asp_protease_2,RVT_1,RT_RNaseH_2,zf-H2C2
	JAEMWC010000177.1:314399_320097	5698	Asp_protease_2,RVT_1,RT_RNaseH_2,Integrase_H2C2
	JAEMWC010000186.1:780857_786533	5676	Asp_protease_2,RVT_1,RT_RNaseH_2,Integrase_H2C2
	JAEMWC010000188.1:783709_792566	8857	Asp_protease_2,RT_RNaseH_2,RT_RNaseH,DDE_Tnp_4,DDE_Tnp_4
	JAEMWC010000192.1:214680_219251	4571	Asp_protease_2,RVT_1,RT_RNaseH_2,Integrase_H2C2
	JAEMWC010000203.1:736222_741257	5035	RVT_1,Integrase_H2C2
	JAEMWC010000216.1:1006658_1012189	5531	TetR_C_24,Asp_protease_2,TetR_C_24,RVT_1,RT_RNaseH_2,Integrase_H2C2,rve

	JAEMWC010000220.1:842107_847392	5285	RT_RNaseH_2,Integrase_H2C2
	JAEMWC010000226.1:460507_466189	5682	Asp_protease_2,RVT_1,RT_RNaseH_2,Integrase_H2C2
	JAEMWC010000229.1:186406_191866	5460	gag-asp_proteas,RT_RNaseH_2
	JAEMWC010000235.1:978019_983565	5546	TetR_C_24,Asp_protease_2,TetR_C_24,RVT_1,RT_RNaseH_2,Integrase_H2C2,rve
	JAEMWC010000249.1:308157_319761	11604	DUF1759,zf-CCHC,zf-CCHC,DUF1758,RVT_1,Peptidase_A17,Integrase_H2C2,rve,DUF5641,gag-asp_proteas,RVT_1,RT_RNaseH_2,Integrase_H2C2
	JAEMWC010000251.1:720777_726403	5626	gag-asp_proteas,RVT_1,RT_RNaseH_2,Integrase_H2C2
	JAEMWC010000262.1:606105_611617	5512	gag-asp_proteas,RVT_1,RT_RNaseH_2
	JAEMWC010000265.1:822409_832945	10536	TetR_C_24,Asp_protease_2,TetR_C_24,Sds3,Lipase_GDSL_2,Exo_endo_phos_2,RVT_1,RVT_1,RT_RNaseH_2,Integrase_H2C2,rve
	JAEMWC010000269.1:531627_540452	8825	Asp_protease_2,Exo_endo_phos,RVT_1,RT_RNaseH_2,Integrase_H2C2
	JAEMWC010000279.1:781842_787021	5179	TetR_C_24,TetR_C_24,RVT_1,RT_RNaseH_2,Integrase_H2C2,rve
	JAEMWC010000283.1:45746_51376	5630	Asp_protease_2,RT_RNaseH_2,Integrase_H2C2
	JAEMWC010000313.1:630598_636296	5698	Asp_protease_2,RVT_1,RT_RNaseH_2
	JAEMWC010000328.1:594606_600162	5556	gag-asp_proteas,RT_RNaseH_2,Integrase_H2C2
	JAEMWC010000329.1:590375_595853	5478	Asp_protease_2,RVT_1,RT_RNaseH_2,zf-H2C2
	JAEMWC010000334.1:272793_278339	5546	Asp_protease_2,RVT_1,RT_RNaseH_2,Integrase_H2C2,rve
	JAEMWC010000350.1:353998_362511	8513	CVNH,Lipase_GDSL_2,Exo_endo_phos,RVT_1
	JAEMWC010000361.1:223382_228932	5550	RVT_1,RT_RNaseH_2,Integrase_H2C2,rve
	JAEMWC010000377.1:294948_300591	5643	RVT_1,RT_RNaseH_2,Integrase_H2C2
	JAEMWC010000378.1:150564_1	5411	RVT_1,RT_RNaseH_2,Integrase_H2C2

55975		
JAEMWC010000389.1:332847_341087	8240	Asp_protease_2,Exo_endo_phos_2,Exo_endo_phos_2,RVT_1,RVT_1,DUF1891,RT_RNaseH_2
JAEMWC010000405.1:581724_587332	5608	Asp_protease_2,RT_RNaseH,Integrase_H2C2
JAEMWC010000420.1:397980_403524	5544	Asp_protease_2,RT_RNaseH_2,Integrase_H2C2
JAEMWC010000427.1:613408_618962	5554	Asp_protease_2,RVT_1,RT_RNaseH_2,Integrase_H2C2,rve
JAEMWC010000457.1:333095_338584	5489	TetR_C_24,Asp_protease_2,TetR_C_24,RVT_1,RT_RNaseH,Integrase_H2C2,rve
JAEMWC010000470.1:436739_442267	5528	TetR_C_24,Asp_protease_2,TetR_C_24,RVT_1,RT_RNaseH_2,Integrase_H2C2,rve
JAEMWC010000475.1:75931_81629	5698	Asp_protease_2,RVT_1,RT_RNaseH_2,Integrase_H2C2
JAEMWC010000475.1:508985_514669	5684	Asp_protease_2,RT_RNaseH_2,Integrase_H2C2
JAEMWC010000493.1:438921_444588	5667	Asp_protease_2,RT_RNaseH_2
JAEMWC010000493.1:612167_617726	5559	TetR_C_24,Asp_protease_2,TetR_C_24,RVT_1,RT_RNaseH_2,Integrase_H2C2,rve
JAEMWC010000495.1:72350_78033	5683	Asp_protease_2,RVT_1,RT_RNaseH_2,RT_RNaseH,Integrase_H2C2
JAEMWC010000495.1:519537_525184	5647	gag-asp_proteas,RVT_1,RT_RNaseH_2,Integrase_H2C2
JAEMWC010000498.1:574590_583488	8898	gag-asp_proteas,RVT_1,RT_RNaseH_2,Integrase_H2C2,zf-H2C2,RVT_1
JAEMWC010000504.1:532113_537702	5589	Asp_protease_2,RVT_1,RT_RNaseH_2,zf-H2C2
JAEMWC010000509.1:130211_138389	8178	Asp_protease_2,RVT_1,RT_RNaseH_2,Integrase_H2C2
JAEMWC010000521.1:134126_139662	5536	TetR_C_24,Asp_protease_2,TetR_C_24,RVT_1,RT_RNaseH_2,Integrase_H2C2,rve
JAEMWC010000526.1:94045_99344	5299	Asp_protease_2,RVT_1,RT_RNaseH_2,Integrase_H2C2
JAEMWC010000535.1:57329_62932	5603	Asp_protease_2,RVT_1,RT_RNaseH_2,zf-H2C2
JAEMWC010000535.1:260245_265922	5677	Asp_protease_2,RVT_1,RT_RNaseH_2

	JAEMWC010000537.1:50721_56195	5474	gag-asp_proteas,RVT_1,RT_RNaseH,Integrase_H2C2
	JAEMWC010000576.1:494774_502591	7817	Asp_protease_2,RVT_1,RT_RNaseH_2
	JAEMWC010000586.1:181806_187356	5550	Asp_protease_2,RVT_1,RT_RNaseH_2,Integrase_H2C2,rve
	JAEMWC010000595.1:171038_175552	4514	RVT_1,RT_RNaseH_2
	JAEMWC010000596.1:226786_232409	5623	Asp_protease_2,RVT_1,RT_RNaseH_2,zf-H2C2
	JAEMWC010000605.1:6172_11695	5523	Asp_protease_2,RVT_1,RT_RNaseH_2,Integrase_H2C2,rve
	JAEMWC010000657.1:418488_423669	5181	RT_RNaseH_2
	JAEMWC010000694.1:146991_152647	5656	Asp_protease_2,RVT_1,RT_RNaseH_2,zf-H2C2
	JAEMWC010000700.1:344948_350643	5695	Asp_protease_2,RVT_1,RT_RNaseH_2,Integrase_H2C2
	JAEMWC010000729.1:141561_149531	7970	TMC,Asp_protease_2,RVT_1,RT_RNaseH_2,Integrase_H2C2
	JAEMWC010000754.1:422874_427914	5040	RVT_1,RVT_1,RT_RNaseH_2,Integrase_H2C2
	JAEMWC010000757.1:160152_165845	5693	Asp_protease_2,RVT_1,RT_RNaseH_2,Integrase_H2C2
	JAEMWC010000760.1:294241_299831	5590	Asp_protease_2,RVT_1,RT_RNaseH_2,Integrase_H2C2
	JAEMWC010000763.1:41873_47400	5527	Asp_protease_2,RT_RNaseH_2,RT_RNaseH,zf-H2C2
	JAEMWC010000770.1:207671_213392	5721	Asp_protease_2,RVT_1,RT_RNaseH_2,Integrase_H2C2
	JAEMWC010000777.1:75060_80596	5536	TetR_C_24,Asp_protease_2,TetR_C_24,RVT_1,RT_RNaseH_2,Integrase_H2C2,rve
	JAEMWC010000780.1:152781_158783	6002	gag-asp_proteas,RVT_1,RT_RNaseH_2,Integrase_H2C2
	JAEMWC010000826.1:34518_40061	5543	RT_RNaseH_2,Integrase_H2C2
	JAEMWC010000831.1:381786_387331	5545	RT_RNaseH_2,Integrase_H2C2
	JAEMWC010000858.1:324863_3	5680	Asp_protease_2,RVT_1,RT_RNaseH_2

	30543		
	JAEMWC010000888.1:2831_12014	9183	Exo_endo_phos_2,RVT_1,RVT_1
	JAEMWC010000915.1:30802_36495	5693	Asp_protease_2,RVT_1,RT_RNaseH_2,Integrase_H2C2
	JAEMWC010000918.1:247501_252948	5447	Asp_protease_2,RVT_1,RT_RNaseH_2,Integrase_H2C2
	JAEMWC010000921.1:315556_321209	5653	gag-asp_proteas,RVT_1,RT_RNaseH_2,Integrase_H2C2
	JAEMWC010000956.1:246165_251619	5454	Asp_protease_2,RT_RNaseH
	JAEMWC010000964.1:60808_66514	5706	Asp_protease_2,RVT_1,RT_RNaseH_2,Integrase_H2C2
	JAEMWC010000970.1:48523_53859	5336	RVT_1,RT_RNaseH_2,RT_RNaseH,Integrase_H2C2,CBP_CCPA
	JAEMWC010000996.1:134624_139833	5209	SpdB,SpdB,RVT_1,Integrase_H2C2,rve
	JAEMWC010001058.1:278632_284161	5529	Asp_protease_2,DMAP_binding,DMAP_binding,RT_RNaseH_2,Integrase_H2C2
	JAEMWC010001062.1:251843_257383	5540	Asp_protease_2,RVT_1,RT_RNaseH_2,Integrase_H2C2,rve
	JAEMWC010001068.1:2343_8285	5942	Asp_protease_2,RVT_1,RT_RNaseH_2,Integrase_H2C2
	JAEMWC010001088.1:155432_161131	5699	Asp_protease_2,RVT_1,RT_RNaseH_2,Integrase_H2C2
	JAEMWC010001089.1:240324_252097	11773	Tropomyosin,Exo_endo_phos_2,RVT_1,zf-RVT
	JAEMWC010001142.1:166972_172456	5484	gag-asp_proteas,RVT_1,RT_RNaseH_2,Integrase_H2C2
	JAEMWC010001143.1:133108_138801	5693	Asp_protease_2,RVT_1,RT_RNaseH_2,Integrase_H2C2
	JAEMWC010001159.1:220271_224950	4679	RVT_1,RT_RNaseH_2,zf-H2C2
	JAEMWC010001196.1:130682_135559	4877	RT_RNaseH_2
	JAEMWC010001197.1:15002_20970	5968	Asp_protease_2,RVT_1,RT_RNaseH_2,Integrase_H2C2
	JAEMWC010001268.1:71665_77069	5404	Asp_protease_2,RVT_1,RT_RNaseH_2,Integrase_H2C2

	JAEMWC010001278.1:111576_117166	5590	Asp_protease_2,RVT_1,RT_RNaseH_2,zf-H2C2
	JAEMWC010001306.1:106398_111451	5053	Asp_protease_2,Integrase_H2C2
	JAEMWC010001317.1:42671_52878	10207	DUF1759,gag-asp_proteas,RVT_1,RT_RNaseH_2,Integrase_H2C2
	JAEMWC010001328.1:146664_152209	5545	Asp_protease_2,RVT_1,RT_RNaseH_2,Integrase_H2C2
	JAEMWC010001335.1:117552_123137	5585	Asp_protease_2,RVT_1,RT_RNaseH_2,zf-H2C2
	JAEMWC010001413.1:186_5166	4980	Asp_protease_2,RVT_1,RT_RNaseH_2
	JAEMWC010001413.1:147145_151981	4836	Asp_protease_2,RVT_1,RT_RNaseH_2
	JAEMWC010001416.1:89451_95683	6232	Asp_protease_2,Integrase_H2C2,Integrase_H2C2
	JAEMWC010001464.1:83104_88645	5541	TetR_C_24,Asp_protease_2,TetR_C_24,RVT_1,RT_RNaseH_2,Integrase_H2C2,rve
	JAEMWC010001468.1:37812_42679	4867	RVT_1,RT_RNaseH_2,Integrase_H2C2
	JAEMWC010001510.1:14312_19908	5596	Asp_protease_2,RVT_1,RT_RNaseH_2,Integrase_H2C2
	JAEMWC010001532.1:124172_130168	5996	gag-asp_proteas,RT_RNaseH_2,RT_RNaseH,zf-H2C2
	JAEMWC010001582.1:76560_82091	5531	Asp_protease_2,RVT_1,RT_RNaseH_2,Integrase_H2C2,rve
	JAEMWC010001586.1:71280_76877	5597	gag-asp_proteas,RVT_1,RT_RNaseH_2,Integrase_H2C2
	JAEMWC010001615.1:113223_118088	4865	RVT_1,RT_RNaseH_2,Integrase_H2C2
	JAEMWC010001635.1:43507_49115	5608	Asp_protease_2,RVT_1,RT_RNaseH_2,Integrase_H2C2
	JAEMWC010001658.1:99093_104609	5516	gag-asp_proteas,RT_RNaseH_2
	JAEMWC010001706.1:59568_65424	5856	Asp_protease_2,RVT_1,RT_RNaseH
	JAEMWC010002084.1:52571_58078	5507	RVT_1,RT_RNaseH_2
Pocillopora verrucosa	JAAVTL010000550.1:119360_134667	15307	

Porites rus	OKRP01000001.1:884814_890875	6061	RT_RNaseH,Integrase_H2C2
Sanderia malayensis	RQOL01000342.1:33303_38735	5432	RVT_1,RT_RNaseH_2
Trachythela sp. YZ-2020	JADLSH010000192.1:2507666_2514169	6503	RVT_1,RT_RNaseH_2,Integrase_H2C2
	JADLSH010000362.1:627221_632671	5450	RVT_1,RT_RNaseH,Integrase_H2C2,rve
	JADLSH010000347.1:227064_232381	5317	RVT_1,RT_RNaseH
	JADLSH010000282.1:1548498_1552634	4136	RVT_1,RT_RNaseH
	JADLSH010000282.1:1950141_1960316	10175	Asp_protease_2,RVT_1,RT_RNaseH_2
	JADLSH010000062.1:4210_9724	5514	Asp_protease_2,RVT_1,RT_RNaseH_2
	JADLSH010000258.1:575304_590814	15510	Asp_protease_2,RVT_1,RT_RNaseH_2,DUF4817
	JADLSH010000043.1:154094_159672	5578	gag-asp_proteas,RVT_1,RT_RNaseH_2,Integrase_H2C2
	JADLSH010000152.1:110885_118824	7939	Exo_endo_phos_2,RVT_1,RVT_1,RT_RNaseH_2,Integrase_H2C2
	JADLSH010000152.1:429411_434864	5453	RVT_1,RT_RNaseH_2,RT_RNaseH,Integrase_H2C2,rve
	JADLSH010000393.1:75877_81242	5365	RVT_1,RT_RNaseH,Integrase_H2C2,Integrase_H2C2,rve
	JADLSH010000393.1:9384936_9390395	5459	gag-asp_proteas,RVT_1,RT_RNaseH_2
	JADLSH010000236.1:1150491_1157867	7376	RVT_1,RT_RNaseH,RVT_1,Integrase_H2C2,rve
	JADLSH010000260.1:4732047_4737547	5500	Asp_protease_2,RVT_1,RT_RNaseH_2,Integrase_H2C2
	JADLSH010000072.1:940033_945683	5650	gag-asp_proteas,RVT_1,RT_RNaseH_2,Integrase_H2C2,ATG11
	JADLSH010000350.1:4737493_4746590	9097	Asp_protease_2,RVT_1,RT_RNaseH_2
	JADLSH010000311.1:2172849_2178414	5565	Asp_protease_2,RVT_1,RT_RNaseH_2
	JADLSH010000311.1:3903687_3909145	5458	Asp_protease_2,RVT_1,RT_RNaseH_2,rve

	JADLSH010000138.1:331855_337236	5381	RVT_1,RT_RNaseH,Integrase_H2C2,rve
	JADLSH010000389.1:2053990_2058938	4948	RVT_1,RT_RNaseH_2
	JADLSH010000389.1:5270451_5275910	5459	RT_RNaseH,Integrase_H2C2,rve
	JADLSH010000387.1:1639343_1646622	7279	Asp_protease_2,RVT_1,RT_RNaseH_2,Integrase_H2C2
	JADLSH010000387.1:3589943_3594350	4407	gag-asp_proteas,Integrase_H2C2
	JADLSH010000387.1:5919925_5925556	5631	gag-asp_proteas,RVT_1,RT_RNaseH_2,Integrase_H2C2
	JADLSH010000271.1:654772_664756	9984	Asp_protease_2,RVT_1,RT_RNaseH_2,Integrase_H2C2
	JADLSH010000367.1:5241317_5250583	9266	RVT_1,RT_RNaseH_2,RT_RNaseH,Integrase_H2C2
	JADLSH010000201.1:373828_379076	5248	RT_RNaseH_2,Integrase_H2C2
	JADLSH010000032.1:402432_408026	5594	Asp_protease_2,RVT_1,RT_RNaseH_2,Integrase_H2C2
	JADLSH010000100.1:1197536_1203123	5587	gag-asp_proteas,RT_RNaseH_2,Integrase_H2C2
	JADLSH010000288.1:163940_169145	5205	RVT_1,RT_RNaseH_2,Integrase_H2C2
	JADLSH010000288.1:348934_353418	4484	RVT_1,RT_RNaseH_2,Integrase_H2C2
	JADLSH010000388.1:262239_270080	7841	RVT_1,RT_RNaseH,Toxin_R_bind_C,Toxin_R_bind_C,RVT_1,Integrase_H2C2,rve
	JADLSH010000177.1:1253102_1258554	5452	RVT_1,RT_RNaseH,Integrase_H2C2,rve
	JADLSH010000025.1:726878_732321	5443	RVT_1,RT_RNaseH,Integrase_H2C2
	JADLSH010000358.1:1445239_1452043	6804	RVT_1,RT_RNaseH_2,Integrase_H2C2
	JADLSH010000358.1:3137402_3141304	3902	Herpes_BLRF2,Herpes_BLRF2,Asp_protease_2,RVT_1
	JADLSH010000199.1:55633_61066	5433	RVT_1,RT_RNaseH_2,RT_RNaseH,Integrase_H2C2
	JADLSH010000157.1:440598_44	5454	RVT_1,RT_RNaseH,Integrase_H2C2

		6052		
		JADLSH010000361.1:325247_339149	13902	Asp_protease_2,Dimer_Tnp_hAT,RT_RNaseH_2,Integrase_H2C2
		JADLSH010000391.1:4369417_4375921	6504	gag-asp_proteas,RVT_1,RT_RNaseH_2,Integrase_H2C2
		JADLSH010000391.1:5937895_5943158	5263	gag-asp_proteas,RVT_1,RT_RNaseH,Integrase_H2C2
		JADLSH010000244.1:595694_599282	3588	RVT_1,RT_RNaseH_2,Integrase_H2C2,rve
		JADLSH010000209.1:1161833_1172965	11132	GIY-YIG
		JADLSH010000209.1:4818329_4823757	5428	Asp_protease_2,RVT_1,RT_RNaseH_2
		JADLSH010000209.1:5958268_5963687	5419	RVT_1,RT_RNaseH,Integrase_H2C2,rve
		JADLSH010000395.1:9749355_9754757	5402	gag-asp_proteas,RVT_1,RT_RNaseH_2,Integrase_H2C2,ATG11
		JADLSH010000395.1:10373524_10379069	5545	gag-asp_proteas,RT_RNaseH_2,Integrase_H2C2,ATG11
		JADLSH010000270.1:69591_75215	5624	gag-asp_proteas,RVT_1,RT_RNaseH_2,Integrase_H2C2
		JADLSH010000390.1:2528688_2534200	5512	RVT_1,RT_RNaseH_2,Integrase_H2C2
		JADLSH010000390.1:10751731_10757143	5412	RVT_1,RT_RNaseH_2,Integrase_H2C2
		JADLSH010000390.1:13073140_13079082	5942	RT_RNaseH_2,Integrase_H2C2
Echinodermata	*Lytechinus pictus*	CM027099.1:19793808_19799696	5888	gag-asp_proteas,RT_RNaseH,Integrase_H2C2
		CM027100.1:34100374_34114182	13808	Asp_protease_2,RT_RNaseH
		CM027100.1:34119025_34131792	12767	Asp_protease_2
		CM027100.1:38054527_38060401	5874	RVT_1,RT_RNaseH_2,Integrase_H2C2
		CM027101.1:720986_727366	6380	Asp_protease_2,RT_RNaseH_2,Integrase_H2C2
		CM027101.1:6103712_6109913	6201	RT_RNaseH_2
		CM027101.1:8501400_8507313	5913	gag-asp_proteas,RVT_1,Integrase_H2C2
		CM027101.1:15204821_15209928	5107	Integrase_H2C2
		CM027101.1:59612296_59618229	5933	gag-asp_proteas,RVT_1,RT_RNaseH_2,RT_RNaseH,Integrase_H2C2
		CM027102.1:7285329_7291245	5916	gag-asp_proteas,RVT_1,RT_RNaseH_2,Integrase_H2C2
		CM027102.1:17963011_17968955	5944	RT_RNaseH,Integrase_H2C2

		CM027103.1:8929517_8938229	8712	Retrotrans_gag,zf-CCHC,RVP_2,RVT_1,RT_RNaseH_2,Integrase_H2C2,rve
		CM027103.1:25827643_25841105	13462	Retrotrans_gag,RVP_2,RVT_1,RT_RNaseH_2,Integrase_H2C2,rve,Exo_endo_phos,RVT_1,DUF1891
		CM027104.1:13731052_13736860	5808	Asp_protease_2,RT_RNaseH_2,Integrase_H2C2
		CM027104.1:34053394_34059676	6282	Asp_protease_2,RVT_1,RT_RNaseH_2,Integrase_H2C2
		CM027107.1:4161385_4167819	6434	Asp_protease_2,RT_RNaseH_2
		CM027107.1:18509635_18515552	5917	RT_RNaseH_2,Integrase_H2C2
		CM027107.1:30163427_30169302	5875	RT_RNaseH_2,Integrase_H2C2
		CM027107.1:33387844_33393716	5872	gag-asp_proteas,RVT_1,RT_RNaseH_2,RT_RNaseH,Integrase_H2C2
		CM027108.1:9611559_9617390	5831	Asp_protease_2,RT_RNaseH_2,Integrase_H2C2,Integrase_H2C2
		CM027108.1:36876350_36882152	5802	Asp_protease_2,RVT_1,RT_RNaseH_2,Integrase_H2C2
		CM027109.1:32799779_32805730	5951	gag-asp_proteas,RVT_1,RT_RNaseH_2,Integrase_H2C2
		CM027109.1:37870163_37879063	8900	Retrotrans_gag,gag-asp_proteas,RVT_1,RT_RNaseH_2,Integrase_H2C2,rve
		CM027111.1:3580226_3586145	5919	RT_RNaseH_2,RT_RNaseH,Integrase_H2C2
		CM027112.1:30917921_30923711	5790	Asp_protease_2,RT_RNaseH_2,Integrase_H2C2
		CM027115.1:31801033_31806843	5810	Asp_protease_2,RT_RNaseH_2,Integrase_H2C2
		CM027116.1:3032882_3038838	5956	gag-asp_proteas,RVT_1,RT_RNaseH_2,Integrase_H2C2
		CM027117.1:29428089_29433898	5809	gag-asp_proteas,RVT_1,RT_RNaseH,Integrase_H2C2
		JADFUK010000493.1:6328_13477	7149	gag-asp_proteas,RVT_1,RT_RNaseH,Integrase_H2C2
Mollusca	*Architeuthis dux*	VCCN01003057.1:1558352_1568537	10185	HTH_psq,RVT_1
		VCCN01005964.1:1909271_1920924	11653	RT_RNaseH_2
		VCCN01006291.1:17375_20585	3210	RT_RNaseH
	Argopecten irradians irradians	SAYR01004977.1:1900678_1906314	5636	Asp_protease_2,RVT_1,zf-H2C2,RT_RNaseH_2,Integrase_H2C2
		SAYR01010770.1:556702_559504	2802	
		SAYR01015957.1:5122445_5133271	10826	gag-asp_proteas,RVT_1,RT_RNaseH_2,RT_RNaseH,Integrase_H2C2
		SAYR01019970.1:437406_441423	4017	RVT_1,RT_RNaseH,Integrase_H2C2
		SAYR01027096.1:343246_348673	5427	gag-asp_proteas,RT_RNaseH_2
		SAYR01027469.1:10693_16258	5565	gag-asp_proteas,RVT_1,RT_RNaseH_2,Integrase_H2C2,Integrase_H2C2
		SAYR01027804.1:1961552_19662	4696	Integrase_H2C2

	48		
	SAYR01031119.1:986609_992125	5516	gag-asp_proteas,RVT_1,RT_RNaseH_2,RT_RNaseH,Integrase_H2C2
	SAYR01031483.1:878159_883791	5632	Asp_protease_2,RVT_1,zf-H2C2,RT_RNaseH_2,Integrase_H2C2
	SAYR01033727.1:274368_280052	5684	gag-asp_proteas,RVT_1,RT_RNaseH_2,Integrase_H2C2
	SAYR01034074.1:902721_908057	5336	gag-asp_proteas,RVT_1,RT_RNaseH,Integrase_H2C2
	SAYR01036727.1:127045_132694	5649	Asp_protease_2,RVT_1,zf-H2C2,RT_RNaseH_2,Integrase_H2C2
	SAYR01037756.1:146075_151553	5478	
	SAYR01040920.1:212731_218201	5470	RT_RNaseH_2,Integrase_H2C2
	SAYR01041854.1:407673_413257	5584	Asp_protease_2,RVT_1,zf-H2C2,RT_RNaseH_2,Integrase_H2C2
	SAYR01043244.1:132168_137725	5557	gag-asp_proteas,RVT_1,RT_RNaseH_2,Integrase_H2C2
	SAYR01045709.1:37291_42789	5498	Asp_protease_2,RVT_1,RT_RNaseH_2,Integrase_H2C2
	SAYR01049271.1:373688_386356	12668	
	SAYR01051422.1:818057_823540	5483	Asp_protease_2,RVT_1,RT_RNaseH_2,Integrase_H2C2
	SAYR01052011.1:247856_253494	5638	gag-asp_proteas,RVT_1,RT_RNaseH_2,Integrase_H2C2
	SAYR01052496.1:138237_143866	5629	gag-asp_proteas,RVT_1,RT_RNaseH_2,RT_RNaseH,Integrase_H2C2
	SAYR01059852.1:103063_112682	9619	RVT_1,zf-H2C2,RT_RNaseH_2,Integrase_H2C2
	SAYR01065535.1:38924_44542	5618	RVT_1,RT_RNaseH_2,Integrase_H2C2
	SAYR01070800.1:434578_440010	5432	RT_RNaseH_2,Integrase_H2C2
	SAYR01072125.1:986174_991778	5604	gag-asp_proteas,RVT_1,RT_RNaseH_2,Integrase_H2C2
Crassostrea hongkongensis	CM027466.1:36255485_36265887	10402	RVT_1,RT_RNaseH_2,Integrase_H2C2,RVP,RVT_1,RT_RNaseH_2,RT_RNaseH,Integrase_H2C2,rve
	CM027468.1:33197138_33209394	12256	RVT_1,RT_RNaseH_2
	CM027469.1:10049974_10058348	8374	RT_RNaseH
	CM027470.1:42209276_42218308	9032	gag-asp_proteas,RT_RNaseH_2,Integrase_H2C2
	CM027472.1:40240120_40245560	5440	RVT_1,RT_RNaseH_2,Integrase_H2C2
	CM027473.1:8881593_8886989	5396	RT_RNaseH_2,Integrase_H2C2
	CM027473.1:28025105_28030568	5463	RVT_1,RT_RNaseH_2,Integrase_H2C2
	CM027473.1:47384139_47395262	11123	gag-asp_proteas,RVT_1,Integrase_H2C2
	VIAA01000040.1:524685_530152	5467	RVT_1,RT_RNaseH_2,Integrase_H2C2
	VIAA01000048.1:88647_98190	9543	HTH_Tnp_Tc3_2,DDE_3,RVT_1,RT_RNaseH,Integrase_H2C2
	VIAA01000065.1:224744_232295	7551	
	VIAA01000077.1:79071_84510	5439	gag-asp_proteas,RT_RNaseH_2,Integrase_H2C2

Cyclina sinensis	JAAONU010000001.1:43245772_43251796	6024	gag-asp_proteas,RVT_1,RT_RNaseH_2,Integrase_H2C2
	JAAONU010000002.1:21503305_21508787	5482	RVT_1,RT_RNaseH_2,Integrase_H2C2
	JAAONU010000011.1:24177833_24183895	6062	RVT_1,RT_RNaseH_2,Integrase_H2C2
	JAAONU010000005.1:31597125_31602964	5839	gag-asp_proteas,RVT_1,RT_RNaseH
	JAAONU010000008.1:31174184_31181528	7344	gag-asp_proteas,RVT_1,RT_RNaseH_2
Dracogyra subfuscus	JAECMU010009595.1:2576_7292	4716	RT_RNaseH_2,Integrase_H2C2
	JAECMU010017556.1:255_5850	5595	SRCR
	JAECMU010038839.1:4526_10055	5529	RVT_1,RT_RNaseH
	JAECMU010088575.1:913_6281	5368	RVT_1,RT_RNaseH_2
	JAECMU010106383.1:1695_7130	5435	Asp_protease_2,RT_RNaseH_2
	JAECMU010115763.1:366_6212	5846	RT_RNaseH_2,zf-H2C2,rve
	JAECMU010120001.1:116_5756	5640	gag-asp_proteas,RT_RNaseH_2,Integrase_H2C2
Dreissena rostriformis	VMBQ01002975.1:37098_42780	5682	RVT_1,RT_RNaseH_2,Integrase_H2C2
Euprymna scolopes	SRIE01002907.1:188368_193349	4981	RT_RNaseH_2,Integrase_H2C2
	SRIE01006744.1:187328_194988	7660	gag-asp_proteas,RVT_1,RT_RNaseH_2
	SRIE01009434.1:1020311_1025868	5557	gag-asp_proteas,RT_RNaseH_2
	SRIE01024348.1:131404_145846	14442	DUF2256,zf-H2C2_2,zf-H2C2_2,zf-H2C2_2,zf-H2C2_2,zf-H2C2_2,zf-H2C2_2,zf-H2C2_2,zf-H2C2_2,zf-H2C2_2,zf-H2C2_2,zf-H2C2_2,zf-H2C2_2,zf-H2C2_2,zf-H2C2_2,RT_RNaseH_2
	SRIE01036102.1:442316_446455	4139	RT_RNaseH_2
	SRIE01040183.1:199341_206130	6789	
	SRIE01046672.1:92855_97567	4712	gag-asp_proteas,RVT_1,RVT_1,RT_RNaseH_2
	SRIE01047123.1:1921521_1929275	7754	
	SRIE01051592.1:1040306_1045943	5637	RVT_1,RT_RNaseH_2
Gigantopelta aegis	CM027790.1:21269004_21275048	6044	Asp_protease_2,RVT_1,RT_RNaseH_2
	CM027791.1:34291100_34296676	5576	gag-asp_proteas,RVT_1,RT_RNaseH_2,Integrase_H2C2,Ac76,Ac76
	CM027791.1:45420567_45424690	4123	RVT_1,RT_RNaseH_2

CM027792.1:17284030_17289634	5604	gag-asp_proteas,RVT_1,RT_RNaseH_2,Integrase_H2C2,DUF3413
CM027792.1:36702387_36708702	6315	Asp_protease_2,RVT_1,RT_RNaseH_2,Integrase_H2C2,rve
CM027792.1:83034809_83040274	5465	RVT_1,RT_RNaseH_2,Integrase_H2C2
CM027793.1:14909498_14915686	6188	Ac76,gag-asp_proteas,RVT_1,RT_RNaseH_2,Integrase_H2C2,Ac76
CM027793.1:27519067_27524467	5400	Asp_protease_2,RVT_1,RT_RNaseH_2,Integrase_H2C2
CM027793.1:57859402_57863729	4327	RT_RNaseH_2,Integrase_H2C2
CM027793.1:59431671_59437232	5561	gag-asp_proteas,RVT_1,RT_RNaseH_2,Integrase_H2C2
CM027793.1:89404432_89410030	5598	gag-asp_proteas,RVT_1,RT_RNaseH_2,Integrase_H2C2
CM027793.1:109923651_109929228	5577	gag-asp_proteas,RVT_1,RT_RNaseH_2,Integrase_H2C2,Ac76
CM027794.1:10108371_10113877	5506	Asp_protease_2,RVT_1,RT_RNaseH_2,RT_RNaseH,Integrase_H2C2,rve
CM027794.1:13090124_13094757	4633	RVT_1,RT_RNaseH_2
CM027794.1:21174092_21187267	13175	gag-asp_proteas,RT_RNaseH_2,RT_RNaseH,zf-H2C2,DUF993,DUF993,DUF993
CM027794.1:24741495_24746833	5338	RT_RNaseH,Integrase_H2C2
CM027794.1:39147298_39152865	5567	gag-asp_proteas,RVT_1,RT_RNaseH_2,Integrase_H2C2
CM027795.1:8575798_8581307	5509	gag-asp_proteas,RVT_1,RT_RNaseH_2,Integrase_H2C2,Ac76,Ac76
CM027795.1:20774493_20780454	5961	gag-asp_proteas,RVT_1,RT_RNaseH_2,Integrase_H2C2,Integrase_H2C2,Ac76
CM027795.1:25045909_25051548	5639	Asp_protease_2,RVT_1,RT_RNaseH_2,Integrase_H2C2
CM027795.1:71213902_71219476	5574	Asp_protease_2,RVT_1,RT_RNaseH_2,Integrase_H2C2
CM027796.1:4331271_4337169	5898	gag-asp_proteas,RT_RNaseH_2,Integrase_H2C2
CM027796.1:11649691_11653240	3549	RVT_1
CM027797.1:15929354_15935862	6508	RVT_1,RT_RNaseH,Integrase_H2C2
CM027797.1:19281114_19286368	5254	RT_RNaseH
CM027797.1:42629258_42635017	5759	RVT_1,RT_RNaseH_2,Integrase_H2C2,rve
CM027798.1:5726320_5731895	5575	gag-asp_proteas,RVT_1,RT_RNaseH_2,Integrase_H2C2
CM027798.1:37720635_37726223	5588	Asp_protease_2,RVT_1,RT_RNaseH_2,zf-H2C2
CM027798.1:56195262_56198199	2937	
CM027798.1:58248366_58253943	5577	Asp_protease_2,RVT_1,RT_RNaseH_2,Integrase_H2C2,Integrase_H2C2,Ac76,Ac76
CM027799.1:6525796_6530074	4278	RVT_1,RT_RNaseH_2,Integrase_H2C2,Ac76
CM027799.1:12596400_12601994	5594	gag-asp_proteas,RVT_1,RT_RNaseH_2,Integrase_H2C2
CM027799.1:61485249_61488863	3614	RVT_1,RT_RNaseH_2,Integrase_H2C2

	CM027799.1:65531701_65537277	5576	gag-asp_proteas,RVT_1,RT_RNaseH_2,Integrase_H2C2
	CM027800.1:30286666_30295159	8493	Asp_protease_2,RVT_1,RT_RNaseH_2,Integrase_H2C2
	CM027801.1:714419_726473	12054	Asp_protease_2,RVT_1,RT_RNaseH_2
	CM027801.1:47250541_47256108	5567	gag-asp_proteas,RVT_1,RT_RNaseH_2,Integrase_H2C2
	CM027801.1:49139418_49147153	7735	Asp_protease_2,RVT_1,RT_RNaseH_2,Integrase_H2C2,rve
	CM027802.1:6601501_6607112	5611	gag-asp_proteas,RVT_1,RT_RNaseH_2,Integrase_H2C2
	CM027802.1:28866043_28870737	4694	gag-asp_proteas,RVT_1,RT_RNaseH_2,Integrase_H2C2,rve
	CM027803.1:5648657_5652070	3413	
	CM027803.1:14977104_14982697	5593	gag-asp_proteas,RVT_1,RT_RNaseH_2,Integrase_H2C2,Ac76,Ac76
	CM027803.1:21788376_21793766	5390	Asp_protease_2,RVT_1,RT_RNaseH_2,Integrase_H2C2
	CM027803.1:46726754_46732454	5700	RT_RNaseH_2
	CM027804.1:8258936_8264486	5550	gag-asp_proteas,RT_RNaseH_2,Integrase_H2C2,Ac76,Ac76
	CM027804.1:20996806_21004827	8021	RVT_1,RT_RNaseH_2,Integrase_H2C2
	JAEHGF010001923.1:33343_38877	5534	Asp_protease_2,RT_RNaseH
	JAEHGF010002106.1:28597_34184	5587	Asp_protease_2,RVT_1,RT_RNaseH_2,Integrase_H2C2,rve,DUF3413
Haliotis laevigata	VKKT01000620.1:187378_191881	4503	RT_RNaseH,zf-H2C2
	VKKT01001831.1:114673_118259	3586	RT_RNaseH_2
	VKKT01002766.1:82981_87729	4748	gag-asp_proteas,RT_RNaseH
	VKKT01003315.1:81151_88536	7385	RVT_1,RT_RNaseH
	VKKT01003798.1:36320_41873	5553	RT_RNaseH
	VKKT01004991.1:60201_67544	7343	Integrase_H2C2,RT_RNaseH_2
	VKKT01007177.1:41365_45011	3646	RT_RNaseH_2,Integrase_H2C2
	VKKT01007782.1:21580_32911	11331	RT_RNaseH_2
	VKKT01014756.1:14206_17025	2819	
	VKKT01016532.1:11812_14037	2225	
Haliotis rubra	QXJH01000114.1:163894_169504	5610	gag-asp_proteas,RVT_1,RT_RNaseH_2,Integrase_H2C2
	QXJH01001343.1:162958_168399	5441	RVT_1,RT_RNaseH,zf-H2C2
	QXJH01000147.1:61782_65471	3689	RT_RNaseH_2,Integrase_H2C2
	QXJH01001495.1:9474_14906	5432	gag-asp_proteas,RVT_1,RT_RNaseH_2,zf-H2C2
	QXJH01000169.1:374225_384616	10391	gag-asp_proteas,RVT_1,RT_RNaseH_2
	QXJH01000189.1:966041_971735	5694	gag-asp_proteas,RVT_1,RT_RNaseH_2,Integrase_H2C2

	QXJH01002018.1:40555_46847	6292	Asp_protease,RVT_1,RT_RNaseH_2,Integrase_H2C2
	QXJH01000209.1:1261305_1267016	5711	RVT_1,RT_RNaseH_2,Integrase_H2C2
	QXJH01000231.1:681495_687182	5687	Asp_protease_2,RVT_1,RT_RNaseH_2,Integrase_H2C2
	QXJH01000251.1:680865_686054	5189	RVT_1,RT_RNaseH_2
	QXJH01000260.1:384396_396190	11794	Macoilin,RVT_1,RT_RNaseH,Phage_int_SAM_4,Phage_integrase
	QXJH01000295.1:557336_563093	5757	RVT_1,RT_RNaseH_2,Integrase_H2C2
	QXJH01000036.1:3336907_3342635	5728	Glycos_transf_2,Glycos_transf_2,RT_RNaseH_2,Integrase_H2C2
	QXJH01000363.1:1027650_1033740	6090	Asp_protease_2,RVT_1,RT_RNaseH,Integrase_H2C2
	QXJH01000488.1:710796_716549	5753	RT_RNaseH
	QXJH01000495.1:683288_690048	6760	Asp_protease_2,RVT_1,RT_RNaseH_2,Integrase_H2C2
	QXJH01000511.1:191885_197376	5491	Asp_protease_2,RVT_1,RT_RNaseH,Integrase_H2C2
	QXJH01000522.1:3436_8492	5056	gag-asp_proteas,RT_RNaseH
	QXJH01000522.1:385969_399842	13873	zf-RING_UBOX,zf-B_box,zf-B_box,RVT_1,RT_RNaseH_2,zf-RING_5,zf-B_box
	QXJH01000066.1:2093813_2097367	3554	RT_RNaseH,zf-H2C2
	QXJH01000690.1:59450_71128	11678	Asp_protease_2
	QXJH01000072.1:1616026_1621766	5740	Asp_protease_2,RVT_1,RT_RNaseH_2,Integrase_H2C2
	QXJH01000738.1:521520_526810	5290	gag-asp_proteas,RT_RNaseH_2
	QXJH01000786.1:413940_419666	5726	gag-asp_proteas,RVT_1,RT_RNaseH_2,Integrase_H2C2
	QXJH01000816.1:46424_52194	5770	Asp_protease_2,RVT_1,RT_RNaseH_2,Integrase_H2C2
	QXJH01000836.1:296007_301468	5461	Asp_protease_2,RVT_1,RT_RNaseH_2,Integrase_H2C2
	QXJH01000863.1:139920_149836	9916	RT_RNaseH_2
	QXJH01000907.1:169251_174409	5158	RVT_1,Integrase_H2C2
	QXJH01000919.1:79692_84828	5136	DUF3679,SH3_5,SH3_5,SH3_5,DUF3679
Haliotis rufescens	QGMO01000002.1:7094194_7099667	5473	RVT_1,Integrase_H2C2
	QGMO01000003.1:11481370_11487048	5678	gag-asp_proteas,RVT_1,RT_RNaseH_2,Integrase_H2C2
	QGMO01000011.1:5335222_5340803	5581	gag-asp_proteas,RVT_1,RT_RNaseH_2,Integrase_H2C2
	QGMO01000015.1:1341892_1347582	5690	RVT_1,RVT_1,RT_RNaseH_2,Integrase_H2C2
	QGMO01000023.1:1703311_1709	5745	RVT_1,RT_RNaseH_2,Integrase_H2C2

	056		
	QGMO01000041.1:230267_235717	5450	RVT_1,RT_RNaseH,Integrase_H2C2,rve
	QGMO01000048.1:2954702_2960430	5728	RVT_1,RT_RNaseH_2,Integrase_H2C2
	QGMO01000057.1:2738767_2744346	5579	Asp_protease_2,RVT_1,RT_RNaseH,Integrase_H2C2,rve
	QGMO01000057.1:3625042_3631950	6908	gag-asp_proteas,RT_RNaseH_2
	QGMO01000076.1:1447512_1453187	5675	gag-asp_proteas,RVT_1,RT_RNaseH_2,Integrase_H2C2
	QGMO01000122.1:1011385_1017100	5715	zf-RVT,gag-asp_proteas,RVT_1,RT_RNaseH_2,Integrase_H2C2
	QGMO01000156.1:937478_949836	12358	Exo_endo_phos_2,RVT_1,RNase_H
	QGMO01000159.1:70738_86462	15724	MFS_1,Fimbrial_PilY2,MFS_1,MFS_1,MFS_1
	QGMO01000160.1:924856_930613	5757	RVT_1,RT_RNaseH_2,Integrase_H2C2
	QGMO01000169.1:1768959_1774583	5624	Asp_protease_2,RVT_1,RT_RNaseH_2,Integrase_H2C2,rve
	QGMO01000198.1:600948_609994	9046	VHS
	QGMO01000241.1:521543_527283	5740	RVT_1,zf-H2C2,RT_RNaseH_2,Integrase_H2C2
	QGMO01000265.1:254420_260102	5682	gag-asp_proteas,RVT_1,RT_RNaseH_2,Integrase_H2C2
	QGMO01000268.1:1345647_1351325	5678	gag-asp_proteas,RVT_1,RT_RNaseH_2,Integrase_H2C2
	QGMO01000295.1:1290415_1296047	5632	gag-asp_proteas,RVT_1,RT_RNaseH_2,Integrase_H2C2
	QGMO01000309.1:573659_579264	5605	Asp_protease_2,RVT_1,RT_RNaseH_2,Integrase_H2C2,rve
	QGMO01000311.1:739277_744424	5147	zf-RVT,gag-asp_proteas,RVT_1,RT_RNaseH_2,Integrase_H2C2
	QGMO01000325.1:1318474_1324138	5664	gag-asp_proteas,RVT_1,RT_RNaseH_2,Integrase_H2C2
	QGMO01000355.1:450184_455749	5565	gag-asp_proteas,RVT_1,RT_RNaseH_2,RT_RNaseH,Integrase_H2C2
	QGMO01000368.1:153234_15884	5610	RVT_1,RT_RNaseH,Integrase_H2C2,SCAMP

	4		
	QGMO01000387.1:267224_271027	3803	ASC,ASC
	QGMO01000388.1:264937_274409	9472	RVT_1,RT_RNaseH_2,Integrase_H2C2
	QGMO01000414.1:626417_631981	5564	gag-asp_proteas,RVT_1,RT_RNaseH_2,Integrase_H2C2
	QGMO01000434.1:574310_579255	4945	Asp_protease_2,RVT_1,RT_RNaseH,Integrase_H2C2,rve
	QGMO01000434.1:916590_921585	4995	RT_RNaseH_2
	QGMO01000443.1:760405_766195	5790	RVT_1,RT_RNaseH_2,Integrase_H2C2
	QGMO01000461.1:13740_19335	5595	gag-asp_proteas,RT_RNaseH_2,Integrase_H2C2
	QGMO01000504.1:737249_742580	5331	Asp_protease_2,RT_RNaseH_2,Integrase_H2C2,rve
	QGMO01000510.1:447952_453467	5515	gag-asp_proteas,RVT_1,RT_RNaseH_2,Integrase_H2C2
	QGMO01000639.1:321000_326576	5576	Asp_protease_2,RVT_1,RT_RNaseH,Integrase_H2C2
	QGMO01000650.1:43464_49148	5684	gag-asp_proteas,RVT_1,RT_RNaseH_2,Integrase_H2C2
	QGMO01000652.1:300414_306004	5590	gag-asp_proteas,RVT_1,RT_RNaseH_2,Integrase_H2C2
	QGMO01000670.1:196899_202386	5487	zf_CopZ,Asp_protease_2,RVT_1,RT_RNaseH_2,Integrase_H2C2
	QGMO01000712.1:335895_341361	5466	DUF4164,gag-asp_proteas,RVT_1,RT_RNaseH
	QGMO01000712.1:367707_374734	7027	gag-asp_proteas,RVT_1,RT_RNaseH_2,Integrase_H2C2
	QGMO01000752.1:196836_202544	5708	gag-asp_proteas,RVT_1,RT_RNaseH_2,Integrase_H2C2
	QGMO01000846.1:253680_258940	5260	gag-asp_proteas,RVT_1,RT_RNaseH_2,Integrase_H2C2
	QGMO01000863.1:147797_153361	5564	gag-asp_proteas,RVT_1,RT_RNaseH_2,Integrase_H2C2
	QGMO01000863.1:200115_210409	10294	Asp_protease_2,zf-RVT
	QGMO01000863.1:269015_277327	8312	RVT_1

	QGMO01000884.1:273178_278843	5665	gag-asp_proteas,RVT_1,RT_RNaseH_2,Integrase_H2C2
	QGMO01000900.1:13864_19631	5767	gag-asp_proteas,RVT_1,RT_RNaseH_2,Integrase_H2C2
	QGMO01000956.1:227891_233399	5508	RVT_1,RT_RNaseH_2,Integrase_H2C2
	QGMO01000960.1:51063_56727	5664	gag-asp_proteas,RVT_1,RT_RNaseH_2,Integrase_H2C2
	QGMO01001046.1:61551_67215	5664	gag-asp_proteas,RVT_1,RT_RNaseH_2,Integrase_H2C2
	QGMO01001132.1:60804_66547	5743	RVT_1,RT_RNaseH,Integrase_H2C2
	QGMO01001133.1:147387_152961	5574	gag-asp_proteas,RVT_1,RT_RNaseH_2,RT_RNaseH,Integrase_H2C2
	QGMO01001394.1:28644_34039	5395	gag-asp_proteas,RVT_1,RT_RNaseH_2,Integrase_H2C2
	QGMO01001600.1:19004_24679	5675	gag-asp_proteas,RVT_1,RT_RNaseH_2,Integrase_H2C2
	QGMO01001636.1:58675_64324	5649	gag-asp_proteas,RVT_1,RT_RNaseH_2,Integrase_H2C2
	QGMO01001817.1:24499_30637	6138	RVT_1,RT_RNaseH_2,Integrase_H2C2
Limacina bulimoides	SWLX010212881.1:5_1633	1628	RVT_1
Limnoperna fortunei	NFUK01000734.1:329526_338759	9233	gag-asp_proteas,RVT_1,RT_RNaseH_2
	NFUK01002330.1:226082_231522	5440	Integrase_H2C2
	NFUK01003095.1:62175_73430	11255	CN_hydrolase,Vanin_C
Lutraria rhynchaena	VIBL01000141.1:133277_138980	5703	RVT_1,RT_RNaseH_2
Magallana hongkongensis	WFKH01003881.1:3215803_3220710	4907	gag-asp_proteas,RVT_1,RT_RNaseH_2
	WFKH01003881.1:69721034_69726349	5315	RT_RNaseH_2,Integrase_H2C2
	WFKH01006014.1:18645611_18651091	5480	gag-asp_proteas,RVT_1,RT_RNaseH_2
	WFKH01006014.1:64928498_64933986	5488	RVT_1,RT_RNaseH,Integrase_H2C2
	WFKH01011801.1:13631499_13636949	5450	RVT_1,RT_RNaseH_2,Integrase_H2C2
	WFKH01011801.1:18768271_18781092	12821	gag-asp_proteas,Voltage_CLC,RVT_1,RT_RNaseH_2,Integrase_H2C2
	WFKH01011925.1:16603455_16608906	5451	RVT_1,RT_RNaseH_2,zf-H2C2
	WFKH01011925.1:62228898_62244189	15291	gag-asp_proteas,RT_RNaseH_2

	WFKH01011925.1:96407036_96412474	5438	RVT_1,RT_RNaseH_2,Integrase_H2C2
	WFKH01011926.1:57978_63349	5371	Integrase_H2C2
	WFKH01011926.1:5456683_5462133	5450	gag-asp_proteas,RVT_1,RT_RNaseH_2,Integrase_H2C2
	WFKH01011926.1:74140975_74149762	8787	gag-asp_proteas,RVT_1,RT_RNaseH_2,Na_trans_assoc
Mercenaria mercenaria	CM025866.1:59011481_59018272	6791	gag-asp_proteas,RT_RNaseH
	CM025866.1:62825995_62831539	5544	gag-asp_proteas,RVT_1,RT_RNaseH_2,Integrase_H2C2
	CM025867.1:100209330_100218908	9578	gag-asp_proteas,RVT_1,RT_RNaseH_2,Integrase_H2C2
	CM025869.1:2315515_2321106	5591	gag-asp_proteas,RVT_1,RT_RNaseH_2,Integrase_H2C2
	CM025869.1:18634340_18639889	5549	gag-asp_proteas,RVT_1,RT_RNaseH_2,Integrase_H2C2
	CM025869.1:88801459_88806986	5527	gag-asp_proteas,RVT_1,RT_RNaseH_2,Integrase_H2C2
	CM025870.1:68328979_68334471	5492	gag-asp_proteas,RVT_1,RT_RNaseH_2,Integrase_H2C2
	CM025870.1:89420574_89428169	7595	gag-asp_proteas,RVT_1,RT_RNaseH_2,Integrase_H2C2
	CM025871.1:80132779_80137986	5207	RT_RNaseH_2,Integrase_H2C2
	CM025872.1:33761943_33767384	5441	gag-asp_proteas,RVT_1,RT_RNaseH_2,Integrase_H2C2
	CM025873.1:28105204_28110783	5579	gag-asp_proteas,RVT_1,RT_RNaseH_2,Integrase_H2C2
	CM025876.1:54422867_54427955	5088	RVT_1,RT_RNaseH_2,Integrase_H2C2
	CM025880.1:55329057_55334625	5568	gag-asp_proteas,RVT_1,RT_RNaseH_2,Integrase_H2C2
	CM025881.1:368695_380017	11322	LNP1,gag-asp_proteas,RVT_1,RT_RNaseH_2,Integrase_H2C2
	CM025881.1:41666549_41672092	5543	gag-asp_proteas,RVT_1,RT_RNaseH_2,Integrase_H2C2
	CM025883.1:5361569_5367111	5542	gag-asp_proteas,RVT_1,RT_RNaseH_2,Integrase_H2C2
Mytilus coruscus	CM029595.1:137619075_137624522	5447	gag-asp_proteas,RT_RNaseH
	CM029596.1:35828935_35838915	9980	gag-asp_proteas,RVT_1,RT_RNaseH_2
	CM029596.1:68463757_68473294	9537	gag-asp_proteas,RVT_1,RT_RNaseH_2
	CM029597.1:85153713_85159429	5716	ELF,ELF,RVT_1,RT_RNaseH_2
	CM029598.1:79205641_79212880	7239	RVT_1,RT_RNaseH_2,Integrase_H2C2
	CM029599.1:81306341_81312116	5775	gag-asp_proteas,RVT_1,RT_RNaseH_2,Integrase_H2C2
	CM029599.1:88579843_88588880	9037	
	CM029601.1:8494681_8500347	5666	RVT_1,RT_RNaseH_2,Integrase_H2C2
	CM029601.1:13718234_13723723	5489	RVT_1,RT_RNaseH_2,Integrase_H2C2
	CM029602.1:2415254_2422421	7167	gag-asp_proteas,RVT_1,RT_RNaseH_2,Integrase_H2C2
	CM029602.1:5617638_5627098	9460	
	CM029602.1:47144619_47150314	5695	Asp_protease_2,RVT_1,RT_RNaseH

	CM029602.1:84653019_84658732	5713	gag-asp_proteas,RT_RNaseH_2
	CM029604.1:93368197_93373238	5041	RVT_1,RT_RNaseH_2,RT_RNaseH
	CM029606.1:24661984_24669005	7021	gag-asp_proteas,RVT_1,RT_RNaseH_2
	CM029607.1:92202_105740	13538	
	CM029607.1:9083150_9088904	5754	gag-asp_proteas,RVT_1,Integrase_H2C2
	CM029607.1:11513521_11520671	7150	Asp_protease_2,RVT_1,RVT_1,RT_RNaseH_2,Integrase_H2C2
	JAASAO010001945.1:89970_99937	9967	RVT_1
Mytilus galloprovincialis	UYJE01000213.1:203953_209759	5806	gag-asp_proteas,RVT_1,RT_RNaseH_2,Integrase_H2C2
	UYJE01000732.1:27514_33278	5764	gag-asp_proteas,RVT_1,RT_RNaseH_2
	UYJE01000976.1:146500_152077	5577	gag-asp_proteas,DUF1356,RVT_1,RT_RNaseH_2
	UYJE01002453.1:204304_210001	5697	gag-asp_proteas,RVT_1,RT_RNaseH_2,Integrase_H2C2
	UYJE01002513.1:39899_45678	5779	gag-asp_proteas,RVT_1,RT_RNaseH_2,Integrase_H2C2
	UYJE01002729.1:7921_19076	11155	RT_RNaseH_2,Integrase_H2C2
	UYJE01002849.1:7595_13351	5756	gag-asp_proteas,RVT_1,RT_RNaseH_2
	UYJE01002919.1:115805_120610	4805	RT_RNaseH_2,Integrase_H2C2
	UYJE01003593.1:36390_42166	5776	gag-asp_proteas,RVT_1,RT_RNaseH_2,Integrase_H2C2
	UYJE01004468.1:118824_124577	5753	gag-asp_proteas,RVT_1,RT_RNaseH,Integrase_H2C2
	UYJE01005312.1:146814_152341	5527	Asp_protease_2,RVT_1,RT_RNaseH_2,Integrase_H2C2
	UYJE01005360.1:82734_88444	5710	gag-asp_proteas,RVT_1,RT_RNaseH_2,Integrase_H2C2
	UYJE01006200.1:130923_143492	12569	RT_RNaseH_2,Integrase_H2C2
	UYJE01006369.1:105383_111072	5689	Asp_protease_2,RT_RNaseH_2,Integrase_H2C2
	UYJE01006538.1:4282_9970	5688	Asp_protease_2,RVT_1,RT_RNaseH,Integrase_H2C2
	UYJE01008673.1:234603_240410	5807	gag-asp_proteas,RVT_1,RT_RNaseH,Integrase_H2C2
	UYJE01008763.1:71508_76377	4869	Asp_protease_2,RVT_1,RT_RNaseH_2,Integrase_H2C2
	UYJE01009011.1:264956_270689	5733	Asp_protease_2,RVT_1,RT_RNaseH_2
	UYJE01009199.1:60698_68295	7597	gag-asp_proteas,RVT_1,RT_RNaseH_2,Integrase_H2C2
	UYJE01010164.1:183052_195369	12317	gag-asp_proteas,RT_RNaseH_2
Ostrea lurida	CAJDSM010003094.1:12_9984	9972	RT_RNaseH_2
	CAJDSM010004777.1:5911_11393	5482	Integrase_H2C2
	CAJDSM010065581.1:4488_10393	5905	
	CAJDSM010147473.1:9950_11096	1146	
Panopea generosa	CADEBB010000002.1:8704166_8709369	5203	Cript,gag-asp_proteas,RVT_1,RT_RNaseH_2,Integrase_H2C2

	CADEBB010000003.1:57054082_57066165	12083	gag-asp_proteas,RVT_1,RT_RNaseH_2,Integrase_H2C2,Exo_endo_phos,RVT_1
	CADEBB010000004.1:63494947_63500370	5423	gag-asp_proteas,RT_RNaseH_2,Integrase_H2C2
	CADEBB010000005.1:58629271_58633350	4079	RT_RNaseH_2,Integrase_H2C2
	CADEBB010000006.1:43787150_43790918	3768	RVT_1,RT_RNaseH_2
	CADEBB010000008.1:35229650_35235147	5497	gag-asp_proteas,dUTPase,RVT_1,RT_RNaseH_2,Integrase_H2C2
	CADEBB010000009.1:7842534_7847370	4836	gag-asp_proteas,RVT_1,RT_RNaseH_2,Integrase_H2C2
	CADEBB010000013.1:25425782_25431635	5853	gag-asp_proteas,RVT_1,RT_RNaseH_2,Integrase_H2C2
	CADEBB010000014.1:42528786_42539511	10725	Exo_endo_phos_2,NTR2,RVT_1,RVT_1,RNase_H,RVT_1,zf-RVT
	CADEBB010000015.1:14773673_14779216	5543	gag-asp_proteas,RVT_1,RT_RNaseH_2,Integrase_H2C2
	CADEBB010000015.1:14877017_14882565	5548	gag-asp_proteas,RVT_1,RT_RNaseH_2,Integrase_H2C2
	CADEBB010000015.1:23469990_23475879	5889	gag-asp_proteas,RVT_1,RT_RNaseH_2,Integrase_H2C2
	CADEBB010000016.1:13222065_13227570	5505	gag-asp_proteas,dUTPase,RVT_1,RT_RNaseH_2,Integrase_H2C2
	CADEBB010000016.1:24323229_24328525	5296	RVT_1,RT_RNaseH_2
	CADEBB010000016.1:24886254_24891174	4920	RVT_1,RT_RNaseH,Integrase_H2C2
Ruditapes philippinarum	CM018522.1:18520976_18529724	8748	
	CM018528.1:38640730_38642637	1907	Integrase_H2C2
	QUSP01000618.1:430048_434674	4626	Asp_protease_2,RVT_1,RT_RNaseH_2
Saccostrea glomerata	PRKT01000029.1:2279276_2284803	5527	Asp_protease_2,RVT_1,RT_RNaseH_2,Integrase_H2C2
	PRKT01000049.1:498580_504107	5527	RVT_1,RT_RNaseH_2,Integrase_H2C2
	PRKT01000095.1:1143408_1148845	5437	gag-asp_proteas,RVT_1,RT_RNaseH_2,Integrase_H2C2
	PRKT01000131.1:802003_808080	6077	Asp_protease_2,RVT_1,RT_RNaseH_2,Integrase_H2C2
	PRKT01000179.1:926721_935634	8913	Bact_transglu_N,Bact_transglu_N,RVT_1,RT_RNaseH_2
	PRKT01000537.1:227695_233003	5308	RVT_1,RT_RNaseH_2

	PRKT01000579.1:24094_30862	6768	RT_RNaseH_2,RT_RNaseH,Integrase_H2C2
	PRKT01000771.1:141936_154663	12727	gag-asp_proteas,RVT_1,RT_RNaseH_2,RT_RNaseH
	PRKT01000771.1:168805_182317	13512	RVT_1
	PRKT01001234.1:41800_46936	5136	gag-asp_proteas,RVT_1,RT_RNaseH_2,Integrase_H2C2
	PRKT01001504.1:58990_71291	12301	gag-asp_proteas,RVT_1,RVT_1,RT_RNaseH_2,Integrase_H2C2,RT_RNaseH_2,rve
	PRKT01001564.1:45970_51230	5260	gag-asp_proteas,RVT_1,RT_RNaseH_2,Integrase_H2C2
Sinonovacula constricta	CM017555.1:84658514_84664018	5504	gag-asp_proteas,RVT_1,RT_RNaseH_2,Integrase_H2C2
	CM017556.1:36802660_36808164	5504	gag-asp_proteas,RVT_1,RT_RNaseH_2,Integrase_H2C2
	CM017557.1:22789000_22794508	5508	RVT_1,RT_RNaseH_2,Integrase_H2C2
	CM017557.1:28884138_28892883	8745	
	CM017557.1:34038528_34043914	5386	Asp_protease_2,RVT_1
	CM017557.1:75494026_75499535	5509	gag-asp_proteas,RVT_1,RT_RNaseH_2,Integrase_H2C2
	CM017557.1:80377530_80383004	5474	gag-asp_proteas,RVT_1,RT_RNaseH_2
	CM017559.1:39314681_39320195	5514	gag-asp_proteas,RVT_1,RT_RNaseH_2,Integrase_H2C2
	CM017559.1:65580379_65585898	5519	gag-asp_proteas,RVT_1,RT_RNaseH_2,Integrase_H2C2
	CM017561.1:59901798_59907257	5459	RVT_1,RT_RNaseH_2,Integrase_H2C2
	CM017562.1:26366469_26371434	4965	Asp_protease_2,RVT_1,RT_RNaseH_2,Integrase_H2C2
	CM017564.1:60116640_60130763	14123	DDE_1
	CM017565.1:53372184_53377555	5371	gag-asp_proteas,RT_RNaseH_2
	CM017566.1:36756897_36769382	12485	zf-RVT,RVT_1,zf-RVT
	CM017566.1:42779704_42785211	5507	gag-asp_proteas,RVT_1,RT_RNaseH_2,Integrase_H2C2
	CM017568.1:29783377_29788243	4866	RT_RNaseH
	CM017568.1:30997786_31003290	5504	gag-asp_proteas,RVT_1,RT_RNaseH_2,Integrase_H2C2
	CM017569.1:15943814_15949338	5524	gag-asp_proteas,RVT_1,RT_RNaseH_2,Integrase_H2C2
	CM017569.1:19276821_19282320	5499	gag-asp_proteas,RVT_1,RT_RNaseH_2,Integrase_H2C2
	CM017571.1:20902774_20908273	5499	gag-asp_proteas,RVT_1,RT_RNaseH_2,Integrase_H2C2
Tegillarca granosa	JABXWC010000001.1:33012469_33017961	5492	gag-asp_proteas,RVT_1,RT_RNaseH_2,Integrase_H2C2
	JABXWC010000001.1:37370648_37375727	5079	Asp_protease_2,RVT_1,RT_RNaseH,Integrase_H2C2,Integrase_H2C2,rve
	JABXWC010000011.1:40318426_40324274	5848	RVT_1,RT_RNaseH_2,Integrase_H2C2
	JABXWC010000012.1:31541565_31547756	6191	Asp_protease_2,RVT_1,RT_RNaseH_2,RT_RNaseH,Integrase_H2C2
	JABXWC010000014.1:22042682_22048169	5487	gag-asp_proteas,RVT_1,RT_RNaseH_2,Integrase_H2C2
	JABXWC010000015.1:32269360_	5524	Asp_protease_2,RVT_1,RT_RNaseH_2,Integrase_H2C2

		32274884		
		JABXWC010000016.1:31868065_31873512	5447	Asp_protease_2,RVT_1,zf-H2C2,RT_RNaseH_2,Integrase_H2C2
		JABXWC010000018.1:24716726_24722248	5522	gag-asp_proteas,RVT_1,RT_RNaseH_2,Integrase_H2C2
		JABXWC010000005.1:311082_316590	5508	Asp_protease_2,RVT_1,RT_RNaseH
		JABXWC010000005.1:33662842_33668284	5442	Asp_protease_2,RVT_1,RT_RNaseH_2,Integrase_H2C2
		JABXWC010000006.1:19260883_19266407	5524	gag-asp_proteas,RVT_1,RT_RNaseH_2,Integrase_H2C2
		JABXWC010000006.1:45953255_45958120	4865	gag-asp_proteas,RVT_1,RT_RNaseH,Integrase_H2C2,rve
		JABXWC010000007.1:24684903_24690510	5607	gag-asp_proteas,RVT_1,RT_RNaseH_2,Integrase_H2C2
		JABXWC010000007.1:38230174_38235673	5499	gag-asp_proteas,RVT_1,RT_RNaseH_2,Integrase_H2C2
		JABXWC010000007.1:45093618_45099134	5516	gag-asp_proteas,RVT_1,RT_RNaseH_2,Integrase_H2C2
		JABXWC010000008.1:13262192_13267652	5460	Asp_protease_2,RVT_1,zf-H2C2,RT_RNaseH_2,Integrase_H2C2
		JABXWC010000008.1:25958327_25963978	5651	Asp_protease_2,RT_RNaseH_2
		JABXWC010000009.1:42913014_42919555	6541	
Nemertea	*Notospermus geniculatus*	NMRB01000172.1:262939_268686	5747	RVT_1,RT_RNaseH_2,Integrase_H2C2
		NMRB01000414.1:307959_313805	5846	Asp_protease_2,RVT_1,zf-H2C2,RT_RNaseH_2,Integrase_H2C2
		NMRB01000756.1:214932_220727	5795	Asp_protease_2,RVT_1,RT_RNaseH_2
		NMRB01001265.1:178119_184240	6121	Asp_protease_2,RVT_1,RT_RNaseH_2,Integrase_H2C2
		NMRB01002570.1:27667_33214	5547	RVT_1,RT_RNaseH_2,Integrase_H2C2
		NMRB01002769.1:54341_59302	4961	RVT_1,RT_RNaseH_2,Integrase_H2C2
Phoronida	*Phoronis australis*	NMRA01000145.1:739613_743166	3553	RT_RNaseH_2,Integrase_H2C2
		NMRA01000168.1:41851_51469	9618	
		NMRA01000198.1:237609_23883	1225	

		4		
		NMRA01000021.1:1613011_1616891	3880	RT_RNaseH_2
		NMRA01000240.1:445213_450206	4993	RT_RNaseH
		NMRA01000252.1:284187_289381	5194	gag-asp_proteas,RVT_1,RT_RNaseH_2,Integrase_H2C2
		NMRA01000305.1:79944_85155	5211	gag-asp_proteas,RVT_1,RT_RNaseH_2,Integrase_H2C2
		NMRA01000331.1:344388_348122	3734	RT_RNaseH_2
		NMRA01000334.1:278436_283524	5088	RVT_1,RT_RNaseH_2,Integrase_H2C2
		NMRA01000373.1:394966_400190	5224	gag-asp_proteas,RVT_1,RT_RNaseH_2,Integrase_H2C2
		NMRA01000411.1:33959_38066	4107	
		NMRA01000619.1:116933_123333	6400	RVT_1,RT_RNaseH,Integrase_H2C2
		NMRA01000637.1:109758_115088	5330	gag-asp_proteas,RVT_1,RT_RNaseH_2,Integrase_H2C2
		NMRA01000071.1:57426_63810	6384	gag-asp_proteas,RT_RNaseH_2,THAP
		NMRA01000897.1:7588_13943	6355	RVT_1,RT_RNaseH_2,Integrase_H2C2,rve
		NMRA01000900.1:89735_105693	15958	RVT_1,Integrase_H2C2
Tunicata	*Halocynthia aurantium*	QXVX01006549.1:8116_14877	6761	RVT_1,RT_RNaseH,Integrase_H2C2
	Halocynthia roretzi	QRAM01000374.1:41049_49767	8718	RT_RNaseH
		QRAM01000757.1:13377_17375	3998	RT_RNaseH_2,Integrase_H2C2
		QRAM01000887.1:1738_7159	5421	gag-asp_proteas
Vertebrata	*Anabarilius grahami*	RJVU01024579.1:2979185_2984096	4911	gag-asp_proteas,RT_RNaseH_2,Integrase_H2C2
		RJVU01069573.1:898552_904801	6249	Asp_protease_2,RVT_1,RT_RNaseH_2,Integrase_H2C2
		RJVU01074764.1:313992_319437	5445	Asp_protease_2,RVT_1,RVT_1,RT_RNaseH,Integrase_H2C2
	Anguilla obscura	CAAKMZ010031743.1:21675_26355	4680	Asp_protease_2,RT_RNaseH_2,Integrase_H2C2
	Anguilla rostrata	LTYT01001347.1:315037_321015	5978	RT_RNaseH_2
	Astyanax mexicanus	NC_035899.1:40914040_40919584	5544	Asp_protease_2,RT_RNaseH,Integrase_H2C2
		NW_019171726.1:189036_19464	5610	Asp_protease_2,RVT_1,RT_RNaseH_2,Integrase_H2C2

	6		
	NW_019172926.1:148456_153371	4915	Integrase_H2C2
Benthosema glaciale	OOFJ01087735.1:2015_7088	5073	gag-asp_proteas,RVT_1,RT_RNaseH_2,Integrase_H2C2
Bregmaceros cantori	OMKY01084078.1:1406_6856	5450	Asp_protease_2,RVT_1,RT_RNaseH_2,Integrase_H2C2
	OMKY01213259.1:2898_6183	3285	Peptidase_M41,Peptidase_M41,Integrase_H2C2
Carassius auratus	NC_039243.1:4959971_4972131	12160	Fibrinogen_C,RVT_1
Clupea harengus	NC_045152.1:3324300_3329963	5663	Asp_protease_2,RVT_1,RT_RNaseH_2,zf-H2C2
	NC_045157.1:8211670_8217002	5332	RT_RNaseH
	NC_045158.1:6731989_6737626	5637	Asp_protease_2,RVT_1,RT_RNaseH_2,Integrase_H2C2
	NC_045160.1:20374097_20381445	7348	DUF4164,Asp_protease_2,RVT_1,RT_RNaseH
	NC_045169.1:23342651_23348284	5633	Asp_protease_2,RVT_1,RT_RNaseH_2,Integrase_H2C2
	NC_045175.1:9253530_9259152	5622	Asp_protease_2,RVT_1,RT_RNaseH_2,Integrase_H2C2
Coilia nasus	CM017714.1:3979351_3984663	5312	gag-asp_proteas,RVT_1,RT_RNaseH,zf-H2C2
	CM017733.1:22651957_22656612	4655	RT_RNaseH_2,RT_RNaseH_2,rve
	CM017715.1:13493314_13498668	5354	gag-asp_proteas,RVT_1,zf-H2C2,RT_RNaseH_2,Integrase_H2C2
Culter alburnus	RXFN01001009.1:122972_126722	3750	Asp_protease_2,RVT_1
Cyprinus carpio	NC_031726.1:738702_741188	2486	
Diplodus sargus	CAEMYA010000307.1:183457_187672	4215	gag-asp_proteas,RVT_1,RT_RNaseH_2
Diretmoides pauciradiatus	CABFOS010139456.1:960_6376	5416	RVT_1,RT_RNaseH_2,Integrase_H2C2
Epinephelus coioides	UNPE01000331.1:5260309_5265087	4778	Integrase_H2C2
Epinephelus fuscoguttatus	AP022695.1:39572071_39576806	4735	gag-asp_proteas,RVT_1,RT_RNaseH_2,Integrase_H2C2
	AP022681.1:12185893_12191294	5401	RVT_1,RT_RNaseH_2,Integrase_H2C2
Epinephelus lanceolatus	NC_046998.1:36383748_36388862	5114	RT_RNaseH_2
Epinephelus moara	CM016908.1:530172_535654	5482	gag-asp_proteas,RT_RNaseH_2,Integrase_H2C2
	CM016910.1:33791905_33796349	4444	RVT_1,RT_RNaseH
	CM016911.1:37000239_37005614	5375	RVT_1,RT_RNaseH_2
Hypophthalmic	LOHW01010963.1:30059_34175	4116	RT_RNaseH_2,Integrase_H2C2

hthys nobilis			
Labeo gonius	NPDK01010293.1:8646_12449	3803	Asp_protease_2,RVT_1
Leuciscus waleckii	FLSR01004887.1:10629571_10635014	5443	RT_RNaseH_2,RT_RNaseH
Megalobrama amblycephala	RXFO01000256.1:65138_69937	4799	gag-asp_proteas,RVT_1,RT_RNaseH_2
	RXFO01001900.1:96661_104983	8322	gag-asp_proteas,RVT_1,RT_RNaseH_2,gag-asp_proteas,RVT_1,RT_RNaseH_2,Integrase_H2C2,gag-asp_proteas
Odontesthes bonariensis	QUZI01000425.1:2957_7908	4951	gag-asp_proteas,RT_RNaseH_2
	QUZI01004805.1:38372_49642	11270	Myb_DNA-bind_5,PNMA,zf-CCHC,Asp_protease_2,RVT_1,RT_RNaseH_2,RVT_1,Integrase_H2C2,rve,RT_RNaseH_2,RT_RNaseH
	QUZI01010799.1:3486_8515	5029	Asp_protease_2,RT_RNaseH_2,Integrase_H2C2
Onychostoma macrolepis	CM022811.1:1393788_1402841	9053	DUF983,RT_RNaseH_2,Integrase_H2C2
Oxygymnocypris stewartii	QVTF01005343.1:180682_185650	4968	Asp_protease_2,RVT_1,RT_RNaseH_2
	QVTF01006432.1:7999_13529	5530	Asp_protease_2,RVT_1,RT_RNaseH_2,Integrase_H2C2
	QVTF01010131.1:791598_801494	9896	Asp_protease_2,RVT_1,RT_RNaseH_2,Integrase_H2C2,MADF_DNA_bdg,DDE_3
	QVTF01018502.1:1026001_1031480	5479	Integrase_H2C2,rve,RVT_1,RT_RNaseH
	QVTF01019650.1:639791_645599	5808	Asp_protease_2,RVT_1,RT_RNaseH_2
	QVTF01019794.1:358275_363784	5509	Asp_protease_2,RVT_1,RT_RNaseH_2,Integrase_H2C2
	QVTF01019937.1:1756992_1766333	9341	Exo_endo_phos_2,RVT_1,DUF1891
	QVTF01021899.1:175222_189574	14352	Cornifin,Asp_protease_2,RT_RNaseH_2
	QVTF01025030.1:210585_216108	5523	Asp_protease_2,RVT_1,RT_RNaseH_2,Integrase_H2C2
Salarias fasciatus	NC_043753.1:21642645_21649974	7329	DUF2381,gag-asp_proteas,RVT_1,RT_RNaseH_2,Integrase_H2C2,rve
Sardina pilchardus	UIGZ01000963.1:31099_36742	5643	Asp_protease_2,RVT_1,RT_RNaseH_2,rve
	UIGZ01002414.1:16037_21657	5620	Asp_protease_2,RVT_1,RT_RNaseH_2,rve
	UIGZ01002469.1:16120_21731	5611	Asp_protease_2,RVT_1,RT_RNaseH_2,Integrase_H2C2,rve
Scyliorhinus canicula	NC_052146.1:65713623_65728791	15168	zf-RVT,Exo_endo_phos
	NC_052150.1:106154704_106169350	14646	RVT_1,RVT_1,DUF1891,Exo_endo_phos
	NC_052156.1:138167042_138175055	8013	
	NC_052158.1:147649260_147655	5754	

	014		
Selene dorsalis	OMNW01011320.1:11381_16684	5303	gag-asp_proteas
Sinocyclocheilus anshuiensis	NW_015540852.1:191912_195272	3360	Asp_protease_2,RVT_1
	NW_015551036.1:106927_110366	3439	
	NW_015555753.1:145706_154785	9079	RVT_1,RT_RNaseH_2,Integrase_H2C2
Sinocyclocheilus grahami	NW_015505546.1:196_5837	5641	Asp_protease_2,RT_RNaseH_2,Integrase_H2C2
	NW_015505644.1:906675_908888	2213	
	NW_015505770.1:219771_229935	10164	
	NW_015505787.1:531732_537190	5458	Asp_protease_2,RVT_1,RT_RNaseH_2
	NW_015505991.1:202325_214021	11696	Asp_protease_2,RVT_1,Integrase_H2C2,THAP
Sinocyclocheilus rhinocerous	NW_015649585.1:121254_126699	5445	Asp_protease_2,RT_RNaseH_2
	NW_015650704.1:297348_308221	10873	RT_RNaseH,Integrase_H2C2,RVT_1,RT_RNaseH_2
Sparus aurata	NC_044199.1:24387505_24394497	6992	zf-CCHC,gag-asp_proteas,RVT_1,RT_RNaseH_2,RT_RNaseH,Integrase_H2C2,rve
Tenualosa ilisha	PYXC01000974.1:3188_9281	6093	
	PYXC01001354.1:3364876_3370511	5635	Asp_protease_2,RVT_1,RVT_1,RT_RNaseH_2,Integrase_H2C2
	PYXC01001496.1:123030_127720	4690	
	PYXC01001496.1:159071_165173	6102	
Trachurus trachurus	LR991633.1:4293731_4299119	5388	gag-asp_proteas,RVT_1,RT_RNaseH_2,Integrase_H2C2
	LR991641.1:8896034_8905667	9633	V-set,gag-asp_proteas,RVT_1,RT_RNaseH_2,Integrase_H2C2

Supplementary Dataset S1. Representative Kuafuorterviruses with LTRs.

Supplementary Dataset S2. Phylogenetic tree of RT proteins from representative retroelements.

Supplementary Dataset S3. The alignment of RT proteins from representative retroelements.

Supplementary Dataset S4. Phylogenetic tree of Kuafuorterviruses, known LTR-like retroelements, and non-LTR retrotransposons for the small dataset.

Supplementary Dataset S5. The alignment of Kuafuorterviruses, known LTR-like retroelements, and non-LTR retrotransposons for the small dataset.

Supplementary Dataset S6. Phylogenetic tree of Kuafuorterviruses, known LTR-like retroelements, and non-LTR retrotransposons for the large dataset.

Supplementary Dataset S7. The alignment of Kuafuorterviruses, known LTR-like retroelements, and non-LTR retrotransposons for the large dataset.
